# Assessment of monocular human pose estimation models for clinical movement analysis

**DOI:** 10.1038/s41598-025-22626-7

**Published:** 2025-11-05

**Authors:** David Rode, Annika Dunkel, Romina Willi, Peter Wolf, Michele Xiloyannis, Robert Riener

**Affiliations:** 1 ETH Zurich, D-HEST, Zurich, Switzerland; 2 Akina AG, Zurich, Switzerland; 3 Schulthess Klinik, Human Performance Lab, Zurich, Switzerland; 4 Medical Faculty, University of Zurich, Zurich, Switzerland

**Keywords:** Biomedical engineering, Computer science, Health care, Orthopaedics

## Abstract

Recent advances in computer vision and learning-based approaches have led to the emergence of markerless human pose estimation as a promising alternative to established motion capturing methods. Markerless methods offer several advantages, including low hardware requirements, affordability, fast setup, and reduced post-processing efforts. However, the accuracy of these methods and the speed at which poses are inferred tend to be lower when compared with marker-based optical motion capturing, which might restrict their range of applications. This study assessed the accuracy, precision, and inference speed of 11 different open source monocular markerless human pose estimators. For this we created Physio2.2M, a dataset comprising 2.2 million RGB frames of 25 unimpaired participants engaged in physical exercise paired with the corresponding ground truth measurements from an optical motion capture system using passive markers. The mean per joint position error between markerless human pose estimators and marker-based optical motion capturing was found to be in the range of 72 to 122 mm in 2D within the image plane and 146 to 249 mm in 3D when considering depth. The knee flexion angle was measured with a mean absolute error of $9.3-21.9^{\circ }$ in 2D and $14.1-25.8^{\circ }$ in 3D. The elbow flexion angle was measured with a mean absolute error of $21.5-28.9^{\circ }$ in 2D and $16.3-26.0^{\circ }$ in 3D. Some of the investigated 2D human pose estimators can achieve accuracies comparable to visual assessments. The inference speed of direct pose estimators ranged between 25 and 200 FPS and 2D-to-3D lifting methods achieved inference speeds of 117 to 9341 FPS. The accuracy and precision varied greatly between different pose estimators and between different image dimensions. This study offers a valuable comparison on the performance of different pose estimators in applications involving physical activities and highlights current limitations.

## Introduction

The fields of sports science^1^ and biomechanics^2^, the design of medical implants^3^ and prostheses^4^ all greatly benefit from methods capable of measuring human movements. Optical motion capturing (OMC) using passive reflective markers attached to the skin is one of the most common measurement methods, besides OMC with active markers, ultrasonic systems, or magnetic trackers. Passive marker OMC is less invasive than fluoroscopy or bone pins, yet offers high accuracy compared to other noninvasive methods^1^. A major drawback of passive marker OMC is the extended preparation time required before measurements can be conducted due to the positioning of cameras surrounding the measurement volume, the calibration of these cameras, the process of determining anatomical landmarks, and the attachment of reflective markers onto these landmarks. When using body models, accuracy is strongly influenced by the correct placement of markers^5^ and soft tissue artifacts^6^, as markers are prone to displacement during movements. The financial costs associated with these systems are considerable, largely due to the number of infrared cameras required and the license fees for the required software. The long setup time, high investment cost, and necessary anatomical knowledge restrict the use of passive marker OMC primarily to research and development and make it impractical in routine clinical use or home applications.

Recent advances in computer vision and learning-based methods have led to the development of markerless human pose estimation (HPE) methods. These methods estimate human posture from images or videos without markers, providing a viable alternative for human motion analysis. HPE methods require less hardware, are more affordable, and are easier to set up than passive marker OMC. Markerless HPE methods can be monocular, using a single camera, or multidirectional, using multiple cameras^7^. Using a single camera reduces hardware needs, simplifies setup, and avoids calibrating multiple cameras, making monocular methods ideal for clinical or home settings, where time and cost are critical. However, monocular methods are less accurate in depth and more sensitive to self-occlusions compared to multidirectional methods^8^.

### Evolution of HPE

**Fig. 1:**
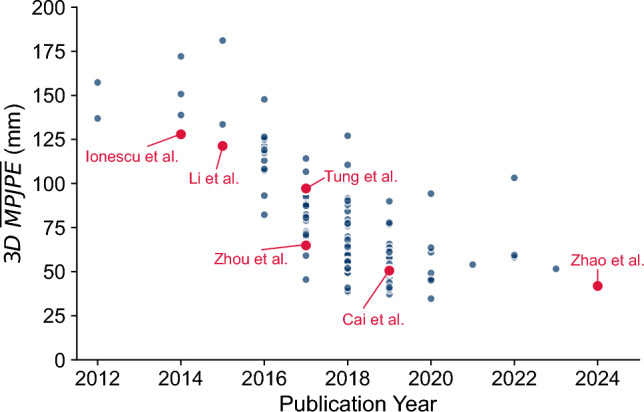
Evolution of the 3D $\overline{MPJPE}$ metric on Human3.6M dataset under Protocol 1 using subjects 9 and 11 for testing with no Procrustes alignment^17^. Selected HPE methods corresponding to different architectures are highlighted in red. A steady increase of the accuracy is noticeable due to improvements in architectures, changes in HPE approaches, and more available training data. Exact values and their sources are listed in Supplementary table S5.

Monocular HPE has been an ongoing topic of research in computer vision for decades, with early methods relying on hand-crafted features to estimate poses^9–11^. Handcrafted features included low-level features such as edges and contours, mid-level features such as Histograms of Oriented Gradients and Scale-Invariant Feature Transform, and high-level representations of body parts or full body structures^12–14^. Using a single feature or a combination of these features, human poses were then estimated using model-based, example-based, or learning-based approaches^14–16^. One such approach proposed by Ionescu et al. used Scale-Invariant Feature Transforms and kernel density estimation to regress poses (see Fig. 1)^17^.

HPE underwent a significant transformation with the emergence of deep neural networks, and in particular convolutional neural networks (CNNs)^11,18^, which surpassed the accuracy of traditional methods^9,13^. Using CNNs for HPE has mostly eliminated the need for hand-crafted features, as they are capable of extracting relevant features directly from raw data^9,14^. DeepPose was the first HPE method to employ a cascade of CNNs to directly regress the joint locations from an image^19^. The authors argued that deep neural networks can capture joint interactions without handcrafted features or explicit pose models. As a single convolutional layer would not have provided sufficiently precise locations, the authors used a cascade of three CNN stages to iteratively refine the joint predictions^19,20^. This cascading approach assumes that a small local context is sufficient to estimate joint locations^21^. Li et al. used a deep CNN for feature extraction and regressed the joint locations from these (see Fig. 1)^22^.

The prediction of probability heatmaps as an intermediary step instead of direct regression was proposed shortly after^23^. The previously mentioned cascaded CNN architecture was combined with the heatmap-based approach in the Convolution Pose Machine, where the stages of CNNs predict heatmaps for each joint^24^. The models were trained using Gaussian probability densities around ground truth joint locations to predict joint probabilities, instead of directly regressing joint locations, improving robustness^12^. The joint locations were then identified by selecting the maximum point from the probability heatmaps. Each stage after the first one considers features from the original input image and the encoded heatmap of the previous stage as input to produce a new heatmap estimate. Due to their large receptive fields, Convolution Pose Machines are capable of learning long-range spatial dependencies between body parts without the need for hand-crafted features. Passing probability heatmaps through convolutional layers increasingly refines them, and the architecture is able to incorporate both low- and high-level features. The authors demonstrated that increasing the size of the receptive field improved the accuracy of joint location predictions. They also showed that the probability heatmaps of easy joints improve the accuracy when estimating difficult joints. Heatmap-based methods have dominated HPE in recent years^16^ and are preferable in many cases^9,20^. The authors noted the robustness of this architecture to nonstandard poses, but identified challenges when multiple humans are present due to difficulties in handling them within a single end-to-end architecture. Tung et al. employed such a convolutional pose machine architecture to extract features and then used an adversarial inverse graphics network to generate 3D poses (see Fig. 1)^25^.

Whereas CPMs used cascaded CNN stages to increase the receptive field to include high-level body features while preserving resolution, other approaches were also proposed to encapsulate high- and low-resolution features. One such approach combined two parallel CNN paths with high and low resolutions^11^. Another approach is based on the hourglass module, which uses downsampling and upsampling operations to capture features of various sizes, such as human orientation, limb arrangement, and joint relations^26^. Downsampling involves repeated convolutional and pooling layers. One branch splits off before each pooling operation and retains the pre-pooling resolution throughout convolutional operations^27^. Meanwhile, the other branch is exposed to pooling operations and downsampled. The upsampling is then achieved by combining features of both branches, which is performed on different size scales by nearest-neighbor interpolation^27^. The use of these skip connections preserves features of all resolution sizes within the hourglass module^27^. Following the favorable results of stacking convolutional layers in Convolution Pose Machines, hourglass modules were also stacked, thereby combining high- and low-resolution spatial features for more context-aware estimations^27^. This model architecture is therefore called the stacked hourglass model^13^. Similarly to preceding work, the final output is a probability heatmap for each joint at every pixel. Repetition of down- and up-sampling between hourglass modules allows intermediate heatmaps to be predicted and supervised, thus training the model to preserve precise spatial locations of joints^26^. Zhou et al. employed a stacked hourglass network to predict 2D poses and a half-hourglass regression for 3D pose estimation (see Fig. 1)^28^.

The pyramid residual module is another architecture designed to capture low- and high-level features, enhancing the model invariance to varying scales of body parts^29^. This module builds upon the hourglass module, but uses different operations at each layer within the hourglass module. In the original hourglass module, the two branches that separated before grouping are transferred to residual modules^27^, leading to a large increase in the output variance when the branches are fused. Using pyramid residual modules instead of residual modules only leads to a small increase in complexity^29^, but increases scale invariance^13^ due to the learned convolutional filters for features of all scales^29^. Similarly to the stacked hourglass architecture, the hourglass modules using pyramid residual modules can also be stacked sequentially, resulting in a cascaded pyramid network^30^. Cai et al. used a cascaded pyramid network to extract 2D poses, which they lifted to 3D by a graph convolutional network (see Fig. 1)^31^.

A drawback of the hourglass and pyramid residual modules is the recovery of high-resolution features from low-resolution features, which may lead to a loss of accuracy^16,32^. High-Resolution Net (HRNet) uses subnetworks of different resolutions in parallel and fuses them repeatedly to achieve more accurate high-resolution features. The use of subnetworks in parallel instead of in series preserves the high-resolution features throughout the model, while repeated fusion across subnetworks exchanges information of different resolutions, resulting in more accurate features. Zhao et al. used a HRNet backbone to extract features and heatmap-guided attention to estimate 3D poses (see Fig. 1)^33^.

All models and architectures mentioned so far focused on single-person HPE^34^, limiting accuracy when multiple humans are present. Multi-person HPE aims at accurately detecting joints in images or videos when multiple humans are visible. Initial methods were two-staged and either bottom-up or top-down. In top-down methods, a detector identifies humans and forms bounding boxes, then applies an HPE method within these boxes^13^. These methods commonly rely on off-the-shelf human detectors^12^, which may have difficulty with partially occluded humans^13^. Furthermore, their run-time is directly proportional to the number of visible humans, which can lead to slow inference speeds if many humans are present in a frame^12,35^. These methods perform better on low-resolution inputs than bottom-up methods, as humans are cropped to uniform scales before being passed to the pose estimator^36^.

Top-down models are sensitive to the accuracy of the human detector due to localization errors and redundancy errors^37^. Inaccurate bounding boxes due to poor localization may lead to poor accuracy or even non-existing poses being estimated. If redundant bounding boxes are proposed for the same human, multiple poses may be estimated despite only one being visible. Methods were proposed to reduce these errors, such as using parametric non-maximum suppression of redundant bounding boxes and training pose estimators to be more robust against inaccurate bounding boxes^37^.

Bottom-up methods first detect all body joints and then group them to form individual human poses^12,13,35^. Their inference speed is more robust to the number of visible humans^36^, as they do not depend on a human detector. This also prevents early commitment errors, which are caused by missing or incorrect bounding boxes and decrease the accuracy of joint locations^34^. However, the assembly of poses is a complex task, which can be computationally expensive^34^ and is influenced by the background and occluded humans^12^. Bottom-up methods require high-resolution inputs to accurately estimate poses if the visible humans have different scales^36^. A proposed method estimates part affinity fields (PAFs) before predicting joint probability heatmaps, allowing it to quickly identify the human that the joint belongs to^34^. PAFs are vector fields that point from a joint center to the next for all pixels within a limb, preserving both position and orientation of the limbs. PAFs encode the relations between body parts of different humans in an image, with each limb having its own PAF. The joint centers are derived from probability heatmaps using non-maximum suppression, and PAFs associate these joints to the correct humans via line integrals over the PAF fields to determine the most confident association. Note that hybrid methods also exist that aim to combine the advantages of top-down and bottom-up approaches by using the bounding boxes of the detectors as an attention mechanism^16^.

Recently, one-stage HPE methods have been developed, which simultaneously perform joint detection and grouping of them into individual poses^12,13^. Their benefits include high and consistent inference speeds as they do not require pre- or post-processing subtasks^35^. A common difficulty for one-stage methods is the presence of humans with different size scales in the input image. This was addressed in Real-Time Multi-person Onestage (RTMO) by using Dynamic Bin Allocation and Dynamic Bin Encoding^35^. This approach dynamically assigns bins to proposed bounding boxes and encodes their positions by sine positional encoding to ensure localized coverage, avoid wasting bins in image areas without visible humans, and reduce quantization errors^35^.

Heatmap-based approaches have dominated the HPE domain ever since they were proposed due to their robustness, accuracy, and easy training^38^. However, they suffer from quantization errors that are especially pronounced for low-resolution input images^38^. Quantization errors occur when continuous coordinates are mapped to discretized heatmaps^38^ because the resolution of these heatmaps affects the accuracy of joint center estimates^7^. Increasing the resolution of heatmaps to counteract this quadratically affects memory and computational cost^7^. Furthermore, heatmap-based models tend to be very large, which limits the type of hardware on which they can run^39^.

The SimCC approach reformulates the HPE problem as separate classification tasks in each image dimension with sub-pixel precision, without the need for time-consuming and resource-intensive refinements or upsampling layers^36,38^. Classification-based approaches can achieve greater accuracy and faster inference speeds than heatmap-based methods, particularly on low-resolution inputs^38^. RTMPose, using SimCC, offers a simple, low-effort architecture that is easily deployable and preserves spatial information by avoiding global pooling in the final classification^36^. Inspired by the success of transformers in computer vision, RTMPose uses a Gated Attention Unit to leverage local and global spatial data and capture joint dependencies^36^. This attention unit is placed between the feature extraction backbone and the joint classifiers^36^.

BlazePose reintroduced another alternative to heatmaps, which uses a regression-based approach due to its efficiency and scalability^39^. It is a two-stage top-down pose estimator and uses a face detector for more robust bounding boxes. The architecture of this model was inspired by the hourglass module, but uniquely predicts heatmaps and directly regresses joint coordinates during training. Both paths use the same encoder but have separate decoders. The heatmaps are used to train the encoder, whereas the gradients of the regression path are not propagated during training. For inference, the heatmap path is removed and only the shared encoder and the direct regression path remain. The result of this is a direct regression hourglass model with an encoder trained on heatmap estimation.

Estimating 3D poses from monocular images presents a serious challenge because it is an ill-posed problem^13^, and training data sets with 3D ground truth labels are scarce^12,13^. The projection of a human pose onto a 2D image results in a loss of information^40^, leading to ambiguity because different poses can appear the same^7,13^. Monocular 3D HPE methods are divided into model-free, which includes direct pose estimators and lifting methods, and model-based techniques^7,12^.

Model-based methods employ parametric human body models like SMPL to estimate 3D poses using optimization or learning-based techniques^12^^7,12^.

Direct methods function similarly to the methods discussed previously. They extract features from monocular inputs, which are then used for heatmap-based, regression-based, or classification-based estimation of 3D joint locations^41^. Lifting methods use 2D pose sequences to predict 3D poses, using models such as fully connected networks, graph convolutional networks, or temporal convolutional networks^7,12,42^. Due to the low memory requirements of 2D poses compared to raw images, lifting methods can use long sequences of poses as input. This allows them to consider long-range temporal dependencies and thus achieve higher accuracy^43^. These methods may struggle in real-time applications, as the lifting process introduces additional delay and extending the receptive field to include future frames considerably increases latency^44^.

Transformer-based methods have become popular for lifting due to their ability to capture correlations in long sequences^45^. For example, the Dual-stream Spatio-Temporal transformer uses cascaded modules with two branches. Each branch includes spatial and temporal self-attention units to capture joint relations within a frame and across frames, respectively. These branches are then adaptively fused^42^. Transformer-based approaches might require a large computational effort to achieve large receptive fields as the computational effort grows quadratically with increasing number of joints and sequence length^43^. Another limitation is the reliance on the preceding pose estimator, which often shows temporal inconsistency and high-frequency noise due to frame-to-frame estimations^43^. One proposed solution to these limitations includes representing long pose sequences in the frequency domain, thus integrating time and frequency features^43^. Sequences of 2D poses are encoded as low-frequency values by a discrete cosine transform before using them as input to the transformer. This reduces high-frequency noise from the 2D HPE step and avoids costly self-attention on all frames of a sequence while retaining large receptive fields^43^. Transformers perform well at capturing long-range relations, whereas graph convolutional networks (GCNs) are especially suited to capture local relations^41^. When used on their own, GCNs tend to perform worse than transformer-based approaches as they mainly focus on local relations^41^. Therefore, a hybrid transformer-graph architecture was proposed, which uses transformers for global dependencies and GCNs for local ones^41^. By integrating features from both, the accuracy of 3D pose estimation improves as the model effectively balances local and global data^41^.

### Motivation

Clinical evaluations^46^, the supervision of physical exercise, and the tracking of therapy progress^47^ are promising applications for monocular HPE. Using monocular HPE could make these measurements more objective, frequent, and feasible for unsupervised home applications due to their affordability and ease of setup compared to other motion capturing methods. However, the accuracy of these methods, especially for physical exercises with complex poses, needs to be considered as well. To date, only few datasets have allowed the comparison of pose estimation methods with a state-of-the-art marker-based OMC in 3D^13^. These include HumanEva^48^, Human3.6M^17^, MPII^49^, COCO^50^, and AMASS^51^, which provide visual data in the form of images or videos along with a ground truth determined by a marker-based OMC system. Many of these existing datasets lack irregular poses^12,42^, making the accuracy of HPE in these contexts uncertain. Currently, there is a lack of datasets with a strong focus on physical exercise, MOYO^52^ and Fit3D^53^ being among the few.

Therefore, the objective of this study was to collect a new dataset containing video recordings of humans performing physical exercise using passive marker OMC as ground truth. We captured Physio2.2M, a new dataset of 25 unimpaired humans performing different physical exercises that comprises over 2.2 million frames. Using this dataset, we evaluated the accuracy, precision, and inference speed of 11 different open source pose estimators, including direct and lifting methods. By including different types of exercises and multiple camera viewing angles, we aimed at creating widely generalizable metrics from dataset averages as commonly done in this field^54,55^. With these metrics, we hope to guide users in choosing pose estimators for applications focused on tracking physical exercise of healthy individuals and aim to highlight their current limitations.

## Methods

### Dataset

**Fig. 2:**
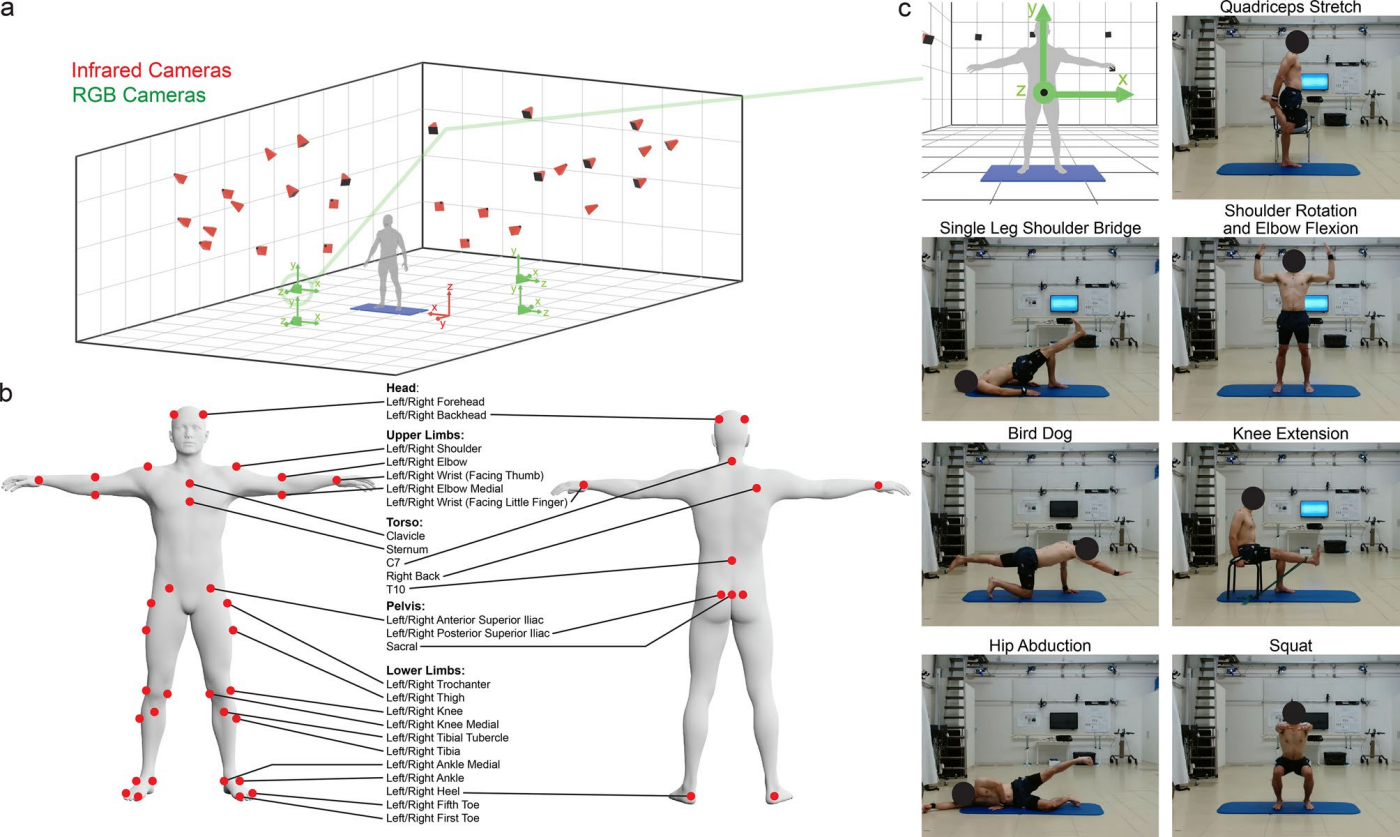
(**a**) Overview over the setup used for simultaneous capturing of passive marker OMC data and RGB videos. We used 27 infrared cameras (red) and 4 RGB cameras (green). The global coordinate system of the passive marker OMC system is indicated in red and the camera-specific coordinates used for error computations are indicated in green. (**b**) Overview over the 46 reflective markers that make up the Plug-in Gait model, a variant of the Conventional Gait Model^56,57^. Participants wore one headband with four markers and two wrist bands with two markers each. The remaining 38 markers were attached to the displayed anatomical landmarks by two experts using double-sided tape. (**c**) Overview over all exercises performed by participants from the perspective of the highlighted RGB camera. ’Shoulder Rotation and Elbow Flexion’ and ’Squat’ were bilateral exercises, all others were unilateral. Each participant performed all exercises for three sets of five repetitions. The camera-specific coordinate system used to compute error metrics for this camera is indicated in the first image.

A dataset of 25 unimpaired participants performing physical exercises was collected for this study (Table 1). Individuals unable to perform physical exercise due to musculoskeletal, neurological, or cognitive deficiencies were excluded. The exercises involved various unilateral and bilateral movements in different positions (Fig. 2b). Each exercise was performed in three sets of five repetitions. The dataset includes videos from four perspectives and motion data from a marker-based OMC system. Data was collected over three months at the Swiss Center for Movement Analysis, Zurich. The study received approval from the Ethics Commission of ETH Zurich (EK 2023-N-4), with all participants giving their informed consent. All experiments were performed in accordance with relevant guidelines and regulations. Written informed consent was obtained from depicted participants for the publication of any identifying images in this manuscript.

**Table 1:** Overview of study participants. Values indicate the mean of attributes with their range in square brackets.

Participants	Age (years)	Height (mm)	Weight (kg)
Male (n=11)	26 [23-31]	1818 [1730-1920]	79 [65-94]
Female (n=14)	26 [20-33]	1649 [1580-1740]	60 [47-80]
Overall (n=25)	26 [20-33]	1724 [1580-1920]	68 [47-94]

### Measurements and analysis

Ground truth was recorded by a passive marker OMC system (Vicon, United Kingdom) with 27 infrared cameras (22 Vicon Vero v2.2, 3 Vicon MX T10, 2 Vicon MX T20-S) at a sampling frequency of 200 Hz. The infrared cameras were arranged to cover the entire measurement area with overlapping views for redundancy and to avoid marker occlusion.

A set of 46 reflective markers (Super-spherical mocap markers, Qualisys AB, Sweden) was used for all measurements (Fig. 2). The Plug-in Gait model (Vicon, United Kingdom), a widely used variant of the Conventional Gait Model^56,57^, was used to reconstruct the joint locations of the hip, knees, ankles, shoulders, elbows, and wrists from the marker locations (Vicon Nexus 2.15, Vicon, United Kingdom)^58^. Participants wore headbands with four markers, wristbands with two markers per hand, and had 38 markers attached to anatomical landmarks by experts using double-sided tape. The infrared cameras were calibrated with a marker wand after reaching operating temperature, and a global reference system was set by placing the wand in fixed ground slots. The reflective surfaces in the measurement space were then masked.

Participants completed a model calibration sequence that involved a 15-second static T-pose and dynamic rotations of the legs, arms, and upper body. During the marker-based OMC measurements, RGB videos were captured at 30 Hz by four webcams (HD C920, Logitech, Switzerland). These cameras were positioned around the participants at two heights and two separate viewpoints. Two cameras were mounted on a stand at heights of 270 mm and 950 mm, respectively. The remaining two cameras were mounted on a stand at identical heights and oriented perpendicular to the other cameras. The participants positioned themselves on an exercise mat located at a distance of 3 m and 3.5 m from the cameras, respectively, and remained at this distance throughout the duration of the measurement. The exercises were performed in a manner that ensured that participants were consistently facing one set of cameras frontally and that the other set of cameras was viewing the sagittal plane. The camera stands were rigid and placed in the exact same position for each measurement. Reflective markers were attached to the cameras to determine their position and orientation in the global reference system.

After starting the OMC and webcam measurements, participants clapped their hands to synchronize both systems. Synchronization was achieved by maximizing the cross-correlation of wrist positions between the two systems. The OMC measurements were downsampled via linear interpolation, reducing the signal frequency from 200 to 30 Hz. The webcams were all connected to the same desktop computer, which read frames from the webcams synchronously at a rate of 30 frames per second (FPS) using OBS Studio (OBS Studio Contributors, open source). The resulting dataset comprised 2.2 million RGB frames with the corresponding 3D joint center locations.

The captured frames were provided as input for the investigated pose estimators. Model inference was performed on the same device (AMD Ryzen 9 7900X, Nvidia RTX 4080, 128 GB RAM) with GPU acceleration (CUDA 12.2, Nvidia, USA) using the Python language (Python 3, Python Software Foundation, open source). Video processing was performed separately for each camera to ensure the independence of the data, as only monocular pose estimators were considered in this study. The investigated lifting methods used the estimated 2D poses to predict 3D pose sequences.

In order to assess the accuracy of markerless pose estimators, we had to represent the estimated poses in the same coordinate frame as the ground truth. By considering the orientation of the cameras within the global coordinate system, we expressed the predicted poses in the same coordinates as the ground truth poses. The estimated poses were translated so that the hip midpoint, also known as the root of the skeleton, coincided with the hip midpoint of the ground truth pose. We then converted pixel units to metric units for poses returned in the image coordinates. The conversion factor was determined by taking the distance from each camera to the ground truth hip midpoint of the participant in each frame and dividing it by the focal length in pixels of each camera. Using these values, we could determine a frame- and camera-specific conversion factor to convert all estimated poses to metric units.

### Pose estimators

In this study, we investigated several different 2D and 3D HPE methods, covering a variety of different architectures and approaches. Openpose was released in 2017 and was the first multi-person real-time capable 2D pose estimator^34^. This method is a bottom-up approach, in which joint locations are initially estimated and human poses are constructed around these using PAFs^55^. Its body pose estimation module was trained on the commonly used COCO and MPII datasets^34^.

Alphapose was published in 2018 and represents a top-down pose estimator capable of detecting multiple humans in an image^59^. This method operates in two stages. Initially, the bounding boxes of humans in an image are determined. Subsequently, the joint center locations within these bounding boxes are predicted^55^. It uses the off-the-shelf YoloV3 detector to detect humans in the input frames and determine their bounding boxes. In this study, we used the ’Fast Pose’ model, which was trained on the Halpe full-body dataset. The architecture of this model uses ResNet50 as the backbone for feature extraction, then employs three dense upsampling convolution modules and a single convolutional layer to generate heatmaps^60^.

In 2019, High-Resolution Net (HRNet) was released, becoming the first human pose estimator to retain high-resolution information through the use of networks in parallel, rather than in series^32^. The outputs of these parallel networks are fused repeatedly, resulting in greater accuracy than with serial connections.^10^. This model also uses YoloV3 as an off-the-shelf detector. We used the HRNet model with a width of 48 in the last three stages of the high-resolution subnetwork, an input size of 384x288 pixels, which was trained on the COCO dataset. This model demonstrated the highest accuracy according to the developers.

Detectron2 was released in 2019 and is a library for object detection that includes a human pose estimation module^61^. The pose estimator is based on the Keypoint R-CNN model architecture, which is based on the Mask R-CNN architecture^62^. It originally was a framework for object instance segmentation, but was trained on the COCO dataset to perform HPE as well to demonstrate its versatility. Training was achieved by treating HPE as a segmentation task, where a pixel corresponding to a joint location was labeled as the foreground and all other pixels as the background. This method is a top-down approach in which masks are estimated for each detected bounding box. Therefore, HPE is effectively treated as a classification task of multiple independent joints without considering spatial relationships between them^62^. We tested the model that uses a 101 layer deep Resnet and Feature Pyramid Network as backbone, which has the highest accuracy according to the developers.

BlazePose is a direct 3D pose estimator, which was first introduced in 2020 and has undergone several updates to date^39^. It is a two-stage top-down method with an hourglass architecture, which uses a novel face detector, a skip-frame detection mechanism, and treats HPE as a regression task to increase its inference speed. It has two different operational modes, called ’World’ and ’Local’. The ’Local’ mode returns the joint center locations in the more common pixel-wise coordinates, whereas the ’World’ mode returns them in metric coordinates with the hip midpoint as the origin. We investigated its three different models, which are designated as ’Lite’, ’Full’, and ’Heavy’ and have different numbers of parameters. The developers trained BlazePose on their own hand-labeled dataset that contains 3D ground truth from the parametric body model GHUM and has a strong emphasis on yoga and fitness poses^63^. Note that the ’Local’ mode delivers joint locations in coordinates that are normalized relative to the dimensions of the input image, and the developers do not provide a clear specification of the normalization constant for the depth coordinate. Consequently, we used the vertical image size to reconstruct the absolute pixel coordinates in depth, which resulted in better results than the more common normalization of the depth by the horizontal image size.

Real-Time Models for Pose Estimation (RTMPose) was released in 2023^36^ and is a two-stage top-down method which treats HPE as two independent classification tasks, following the SimCC approach. This model has three preset modes, which were investigated in this study: ’Balanced’, ’Performance’, and ’Lightweight’. Despite varying input sizes for detectors and pose estimators, all modes share the YoloX detector and the same model architecture. The models use CSPNeXT as a feature extraction backbone, use a transformer to capture dependencies between the joints, and employ two parallel joint coordinate classifiers. RTMPose uses a skip-frame detection mechanism inspired by BlazePose to increase the inference speed, where the detection substep is only rerun after a certain number of frames. The models were trained on seven different datasets, including COCO and MPII. Based on this model, the developers released two further models: the Real-Time Multi-person One-stage (RTMO) model in 2024, and the Real-Time Multi-person Whole-body (RTMW) model in 2024.

RTMO is a one-stage pose estimator designed for the fast inference of multiple poses in a frame^64^. It uses the SimCC dual classification approach to HPE with a YOLO-inspired CSPDarknet backbone. The backbone provides spatial features from which pose features are extracted and refined by a Gated Attention Unit. Bounding boxes are generated from pose features, discretized using Dynamic Bin Allocation, and encoded using Dynamic Bin Encoding^64^. These bounding boxes are then used with the extracted pose features to predict two 1-D heatmaps for all joint probabilities following the SimCC approach. We tested the ’Balanced’, ’Performance’, and ’Lightweight’ models, which have the same input sizes and architectures but differ in number of parameters.

RTMW is a two-step top-bottom whole-body pose estimator, capable of estimating the positions of the face, hands, body, and feet. Its architecture is based on RTMPose and includes a Part-Aggregation Feature Pyramid Network and a High-Efficiency Multiscale Feature Fusion module between the feature extraction backbone and the parallel classification paths to further increase the feature resolutions^65^. As in RTMPose, the investigated models were ’Balanced’, ’Performance’, and ’Lightweight’, all of which use the YoloX detector in the first subtask and differ in their input sizes. These models were trained on a combination of 14 different datasets, including COCO-Wholebody and MPII.

In addition to these direct HPE methods, we also investigated lifting methods in this study, which used the 2D pose sequences generated by the pose estimators mentioned above as input. MotionBERT was released in 2023 and uses cascades of dual-stream spatiotemporal transformers to elevate 2D poses to 3D representations by considering the interactions of joints between frames and within a single frame^42^. The developers used AlphaPose to generate 2D input pose sequences and trained MotionBERT on Human3.6M, AMASS, PoseTrack, and InstaVariety. We tested the MotionBert ’Lite’ model, which uses sequence lengths of 243 frames.

PoseFormerV2, released in 2023, represents an update of the PoseFormer transformer, which was first introduced in 2021^43^. It transforms 2D poses into the frequency domain using the discrete cosine transform, which increases computational efficiency and robustness to noise^41^. Additionally, it is the sole pose estimator in this study, apart from BlazePose ’Global,’ which returns landmarks in metric units. We tested the model, which takes input sequences of 243 frames and was trained on Human3.6M. The developers propose using HRNet to generate the 2D input pose sequences.

The MotionAGFormer model was developed in 2024 and is capable of lifting an entire sequence of 2D poses to 3D^41^. This distinguishes it from the majority of transformers, which are designed to lift only a single pose from a sequence of 2D poses. It integrates a transformer-based approach and a graph-convolutional network, which are adaptively fused together. The combination of these methods and its lightweight architecture makes it a highly resource-efficient approach. We tested the ’Big’ model with input sequence length of 243 frames, which was trained on Human3.6M.

### Metrics for assessment

We represented the joint locations of both measurement systems in a common coordinate system to quantify the distances between them. Each error vector points from the joint location predicted by markerless pose estimation to the joint location measured by the OMC system. We express these error vectors in a camera-specific coordinate system with horizontal, vertical, and depth components, which we denote by *x*, *y*, and *z*.

We define the horizontal component of the error vector for a joint *j* in frame *i* as:

1 $$\begin{aligned} e_{x}^{i,j} = x_{HPE}^{i,j} - x_{OMC}^{i,j} \end{aligned}$$

The vertical and depth components follow accordingly. The error vector has two or three components, depending on the dimension of the estimated pose. For 2D pose estimators, we excluded the depth error.

The position error (PE) of joint *j* is the Euclidean length of its error vector within a single frame. It can be calculated in frame *i* for any joint *j* as:

2 $$\begin{aligned} PE^{i,j} = \sqrt{(e_x^{i,j})^2 + (e_y^{i,j})^2 + (e_z^{i,j})^2} \end{aligned}$$

We compared 12 joint centers common to all pose estimators and the marker-based OMC system: left and right wrist, elbow, shoulder, hip, knee, and ankle. The mean per joint position error ($MPJPE^{i}$) of a pose in frame i is the average PE of these 12 joints:

3 $$\begin{aligned} MPJPE^{i} = \frac{1}{N_{Joints}} \cdot \sum _{j=1}^{N_{Joints}}PE^{i,j} \end{aligned}$$

The joint-averaged absolute error in the horizontal image direction is defined for frame i as:

4 $$\begin{aligned} e_{x}^{i} = \frac{1}{N_{joints}}\sum _{j=1}^{N_{Joints}} |e^{i,j}_{x} |\end{aligned}$$

The joint-averaged errors in vertical and depth direction follow accordingly. Based on these, we calculated the frame- and joint-averaged absolute error in an image direction by taking the mean of the absolute error in a direction over all 12 joints in a frame and then averaging this over all detectable frames, considering all participants, exercises, and camera angles. The mean absolute error in horizontal image direction $\overline{e_{x}}$ is defined as following, with the mean absolute errors in vertical and depth image directions $\overline{e_{y}}$ and $\overline{e_{z}}$ following accordingly:

5 $$\begin{aligned} \overline{e_{x}} = \frac{1}{N_{Frames}} \sum _{i=1}^{N_{Frames}} e_{x}^{i}=\frac{1}{N_{Frames}} \sum _{i=1}^{N_{Frames}} \frac{1}{N_{Joints}}\sum _{j=1}^{N_{Joints}} |e^{i,j}_{x} |\end{aligned}$$

We took the average of the frame-specific $MPJPE^{i}$ over all frames of the dataset, considering all participants, exercises, and camera angles to compute the frame-averaged mean per joint position error:

6 $$\begin{aligned} \overline{MPJPE} = \frac{1}{N_{Frames}} \cdot \sum _{i=1}^{N_{Frames}}MPJPE^{i} \end{aligned}$$

For 2D pose estimators, we did not consider the depth error $e_z$ and the averaged depth error $\overline{e_{z}}$. The $\overline{MPJPE}$ can still be computed from the horizontal and vertical errors alone, and in this case we refer to it as $2D \ \overline{MPJPE}$. If a depth estimation was considered for the computation, we refer to it as $3D \ \overline{MPJPE}$.

The Procrustes-aligned mean per joint position error $PAMPJPE^{i}$ is a useful metric to assess the accuracy of HPE methods at detecting specific joints. It reduces the effect of any scaling errors that might arise from conversion of pixel-wise coordinates to metric ones. Furthermore, it reduces the systematic influence of the hip midpoint on the overall accuracy. This metric uses the generalized orthogonal Procrustes operation, which is solved by singular value decomposition. Procrustes alignment provides the optimal isomorphic scaling ratio, orthogonal rotation matrix, and translation vector such that the set of joints estimated by HPE best matches the set of positions determined by OMC^66^. Because this operation preserves shape, it translates and rotates the joint centers but preserves the angles between them^67^. The Procrustes-aligned errors are therefore a useful metric for fairly comparing the errors of specific joints with other measurement methods. We used MATLAB’s Procrustes function (R 2023a, Mathworks, USA) with scaling and no reflections to preserve pose orientation.

After applying the Procrustes alignment, we computed the Procrustes-aligned position errors $PAPE^{i,j}$ for each joint *j* in frame *i*, similar to equation 2. We refer to the frame-wise average of this joint-specific error as $2D \ \overline{PAPE}^j$ or $3D \ \overline{PAPE}^j$, depending on the dimension of the pose. The Procrustes-aligned mean per joint position error $\overline{PAMPJPE}$ can be computed from the joint-averaged errors $PAPE^{i}$, following equation (6):

7 $$\begin{aligned} \overline{PAMPJPE} = \frac{1}{N_{Frames}} \cdot \sum _{i=1}^{N_{Frames}}PAMPJPE^{i} = \frac{1}{N_{Frames}} \cdot \sum _{i=1}^{N_{Frames}}\frac{1}{N_{Joints}} \cdot \sum _{j=1}^{N_{Joints}}PAPE^{i,j} \end{aligned}$$

We also determined the flexion angles of the elbow and knee joints for comparison between the different methods. We assumed the elbow and knee joints to behave as hinge joints with a single degree of freedom, allowing us to compute them for both HPE and OMC with the scalar product formula.

We computed the mean absolute error ${MAE}^{i,\alpha }$ for joint flexion angle $\alpha$ in frame *i* by considering the corresponding joint flexion angles $\alpha _j^i$ on both sides of each participant:

8 $$\begin{aligned} {MAE}^{i,\alpha } = \frac{1}{2} \cdot ( \ |\alpha _{HPE}^{i,left \ joint} - \alpha _{OMC}^{i,left \ joint} |+ |\alpha _{HPE}^{i,right \ joint} - \alpha _{OMC}^{i,right \ joint} |\ ) \end{aligned}$$

From this we determined $\overline{MAE}^{\alpha }$, which represents the average over the entire dataset and therefore all participants, exercises, and camera orientations for flexion angle $\alpha$:

9 $$\begin{aligned} \overline{MAE}^{\alpha } = \frac{1}{N_{Frames}} \cdot \sum _{i=1}^{N_{Frames}} {MAE}^{i,\alpha } \end{aligned}$$

For 3D pose estimators, we determined the angles using the scalar product of the 3D vectors between the joint centers and denote the resulting error by $3D \ MAE$. 2D pose estimators cannot measure depth, so they cannot determine joint flexion angles unless the vectors are only within the image plane. Therefore, we also compared the projection of the flexion angles for all pose estimators and denote this error as $2D \ MAE$. This ensured a fair comparison, as 2D pose estimators are only capable of measuring the projection of the flexion angle onto the image plane.

Most pose estimators did not detect a pose in some frames, and we excluded these frames from the computation of error metrics. Instead, we report the ratio of detected poses to total poses. For 3D lifting methods, we report the final detection ratio after running the pose estimator and lifting method on it. We measured inference speed by timing each inference step of a given pose estimator and determining the average speed over the entire dataset. In the case of 3D lifting methods, we only report the inference speed of the lifting step.

## Results

### Two-dimensional pose estimators

**Fig. 3:**
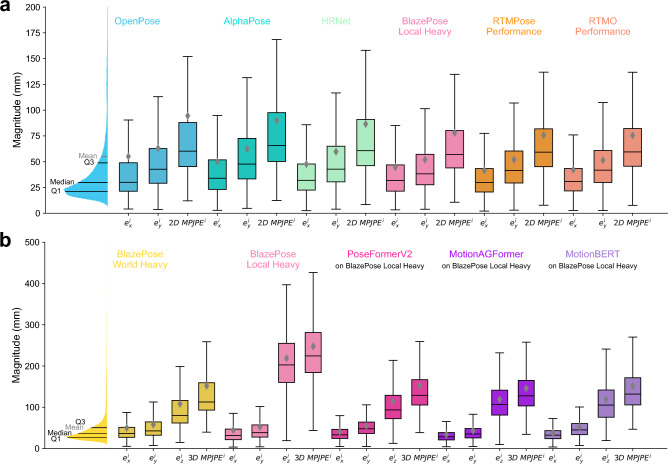
Accuracy and precision of selected pose estimators. The boxplots depict the mean absolute errors in different image directions and the average 2D and 3D mean per joint position errors. These values were averaged over the entire dataset and therefore over all participants, exercises, and camera angles. Only detected frames are considered, and the ratio of detected poses varies significantly between pose estimators. Triangles represent mean values, solid lines indicate median values, and the whiskers include values within 1.5 times the interquartile range. Outliers beyond the whiskers are not depicted. (**a**) 2D pose estimators: The metrics for BlazePose were computed by not considering its depth estimates. Note that the mean absolute error in y-direction is higher than in x-direction for all investigated pose estimators. (**b**) 3D pose estimators: Depth errors are notably larger than in-plane errors for 3D pose estimators.

**Fig. 4:**
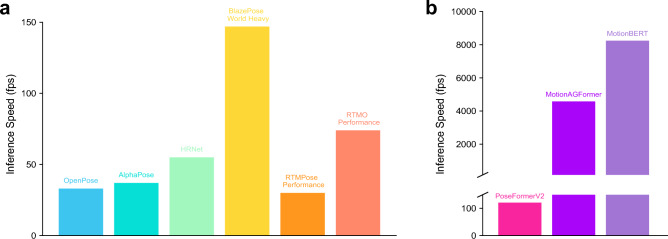
Inference speed of selected direct pose estimators and 2D-to-3D lifting methods. The bars indicate the average measured inference speed over the entire dataset, including all participants, exercises, and camera angles. All models were run with GPU acceleration on the same hardware: AMD Ryzen 9 7900X, Nvidia RTX 4080, 128 GB RAM. (**a**) Direct methods: BlazePose achieves very high inference speeds due to its skip-frame detector and a regression-based approach. RTMO achieves high speeds as it is a one-stage method. (**b**) 2D-to-3D Lifting methods: These methods take 2D pose sequences as input, which are smaller than images and result in very high inference speeds as displayed by MotionBERT and MotionAGFormer.

**Fig. 5:**
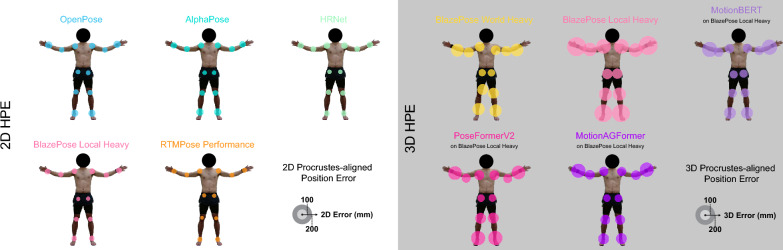
Depiction of estimated joint locations and average joint errors of selected pose estimators. The joints depicted are left and right wrists, elbows, shoulders, hips, knees, and ankles. The circles are centered around the estimated joint position for the depicted frame. Their radius corresponds to the mean Procrustes-aligned joint specific error $\overline{PAPE}^{j}$ in 2D and 3D respectively. These errors were computed by performing Procrustes-alignment of all poses and then taking the average of the joint-specific position error. Notice the smaller errors for the hips and higher errors for wrists and ankles. See Supplementary tables S2 and S4 for exact values.

In total, we evaluated 18 different 2D pose estimation models. The measured $2D \ \overline{MPJPE}$ ranged from 72 to 122 mm (Fig. 3a). The $2D \ \overline{MAE}$ for the knee flexion angle ranged from 9.3 to $21.9^{\circ }$ and from 21.5 to $28.9^{\circ }$ for the elbow flexion angle (Supplementary table S1). The inference speed varied from 25 to 200 FPS (Fig. 4a), with pose detection ratios between 56.50% and 100% (Supplementary table S1). Most pose estimators showed the lowest Procrustes-aligned position error in the hip (Fig. 5), except RTMPose and its derivative RTMO, which were more accurate in the knee (Supplementary table S2).

RTMPose ’Performance’ was the most accurate direct 2D pose estimator with a $e_x$ of 37 mm, $e_y$ of 52 mm, and $2D \ \overline{MPJPE}$ of 72 mm. The $2D \ \overline{PAMPJPE}$ was 53 mm, knee flexion $2D \ \overline{MAE} 9.3^{\circ }$, and elbow flexion $2D \ \overline{MAE} 24.9^{\circ }$. RTMPose predicted a pose in all frames at an inference speed of 30 FPS.

Note that lifting methods can enhance the accuracy of 2D poses in some cases. The overall highest 2D accuracy was displayed by MotionAGFormer when lifting the 2D poses by BlazePose ’Local’ with the ’Heavy’ model. This approach showed a $e_x$ of 34 mm and $e_y$ of 43 mm, leading to a $2D \ \overline{MPJPE}$ of 61 mm on the dataset. The $2D \ \overline{PAMPJPE}$ of this model was measured as 46 mm, the $2D \ \overline{MAE}$ for knee flexion was $8.5^{\circ }$ and $22.1^{\circ }$ for elbow flexion. MotionAGFormer was able to predict a pose for every frame by filling all encountered gaps at an average inference speed of 4580 FPS.

### Three-dimensional pose estimators

In total 26 different 3D pose estimation models were tested. The measured $3D \ \overline{MPJPE}$ ranged from 146 to 249 mm (Fig. 3b). The $3D \ \overline{MAE}$ for knee flexion and elbow flexion ranged from 14.1 to $25.9^{\circ }$ and 16.3 to $26.0^{\circ }$, respectively (Supplementary table S3). The inference speed was between 117 and 9339 FPS (Fig. 4), with detected pose ratios ranging between 14.40% and 100% (Supplementary table S3). All 3D pose estimators had the smallest 3D Procrustes-aligned joint position error in the hip joint (Fig. 5 and Supplementary table S4).

BlazePose ’World’ with the ’Heavy’ model was the best performing direct pose estimation model, achieving a $e_x$ of 50 mm, a $e_y$ of 58 mm, and $e_z$ of 108 mm, leading to a $3D \ \overline{MPJPE}$ of 146 mm. It showed a $3D \ \overline{PAMPJPE}$ of 110 mm, $3D \ \overline{MAE}$ for knee flexion and elbow flexion of $17.2^{\circ }$ and $18.3^{\circ }$. This model detected 98.8% of all poses with an inference speed of 147 FPS.

Lifting the 2D poses of BlazePose ’Local’ with the ’Heavy’ model with MotionAGFormer was the most accurate 3D pose estimation method. This approach showed a $e_x$ of 34 mm, a $e_y$ of 43 mm, and a $e_z$ of 119 mm, leading to a $3D \ \overline{MPJPE}$ of 146 mm. The $3D \ \overline{PAMPJPE}$ of this model was measured as 105 mm, the $3D \ \overline{MAE}$ for knee flexion was $16.7^{\circ }$ and $16.3^{\circ }$ for elbow flexion. This method estimated 100% of all poses with an average inference speed of 4580 FPS.

## Discussion

This study evaluated the accuracy and inference speed of different 2D and 3D pose estimators on a newly captured dataset, which focused on unimpaired individuals performing physical exercise. Accuracy and speed are crucial metrics for evaluating the usability of these methods in real-world applications like clinical measurements, exercise supervision, and gait analysis. The objective of this study was to determine the general accuracy and inference speed of pose estimators for physical exercises and different camera orientations.

### Accuracy of HPE methods

Joint angle errors should be lower than $5^{\circ }$ to allow correct clinical interpretation^68^, which none of the pose estimators tested was able to achieve. However, the accuracy of HPE methods can be compared to visual assessments, which are commonly performed in clinical practice and physical therapy^69,70^. Visual measurements of knee flexion angles during running by human raters led to errors between -54 and $21^{\circ }$ compared to marker-based OMC^71^. The resulting MAE was extracted from the provided figure and was $20.6^{\circ }$. Furthermore, the authors mention poor consistency among the observers, even though they were experienced^71^. The 2D pose estimators investigated in this study showed a MAE in measuring the projected knee flexion angle between 9.3 and $21.9^{\circ }$, and the 3D pose estimators showed a MAE between 14.1 and $25.8^{\circ }$. The majority of investigated 3D pose estimators outperform visual assessments of knee flexion angles in terms of accuracy.

A study on the accuracy of novices in evaluating elbow flexion angles found that only about 16.8 % of the participants rated angles with significant correlation^70^. Another study compared the accuracy of the visual assessments by expert raters with an OMC system and found an MAE of $8.4^{\circ }$ for large flexion angles and $12.2^{\circ }$ for small flexion angles^72^. The 2D pose estimators investigated in this study showed a MAE in measuring the projected elbow flexion angle between 21.5 and $28.9^{\circ }$, and the 3D pose estimators showed a MAE between 16.3 and $26.0^{\circ }$. Currently, HPE methods struggle to accurately measure elbow flexion angles and are outperformed by visual inspection.

Visual assessments tend to display low inter-rater agreements and more bias than alternative measurement methods. Comparisons of visual assessments and clinical goniometers found visual assessments to be of comparable accuracy when performed by experienced experts, but noted that inter-rater agreement was poor especially for inexperienced raters and measurements should be taken by the same rater, which is rarely feasible in clinical settings^69^. The inter-observer reliabilities for elbow flexion angles ranged from 0.38 when comparing an inexperienced rater and an expert surgeon to 0.96 when comparing two expert raters^69^. Meanwhile, visual assessments of the knee flexion angle resulted in an ICC of 0.82 to 0.97^73^. Another study on knee flexion angle measurement found that visual estimation was the least reliable compared to different types of goniometers and described visual estimations as ineffective for documentation and when involving different raters^74^. Using HPE could improve the reliability of clinical measurements while keeping costs low and measurement time short.

All investigated pose estimators showed a higher mean absolute error in the vertical direction than in the horizontal direction. In a subsequent analysis, we found that the vertical errors are much greater than the horizontal errors when viewing the participants in the frontal plane. When viewing the sagittal plane, the horizontal and vertical errors tended to be more similar. As we recorded the participants with the same number of cameras in the frontal and sagittal plane during each exercise, the averaging over the entire dataset led to larger mean absolute vertical errors.

All methods capable of 3D pose estimation exhibited a significantly greater depth error than within the image plane. The mean absolute depth error is approximately two to three times greater than the mean absolute horizontal and vertical errors for all pose estimators investigated. This causes a significant difference between the observed $2D \ \overline{MPJPE}$ and $3D \ \overline{MPJPE}$ values (Supplementary table S3). The errors in depth estimation were due to self-occlusion and the inherent physical limitation of monocular depth estimation. Due to the experimental setup, two cameras were always experiencing significant self-occlusion, i.e., the right legs were self-occluded during the squat exercise and the right upper extremities were self-occluded during the shoulder rotation and elbow flexion exercise when viewing the sagittal plane. In case of no self-occlusion the depth estimation errors can be largely attributed to the ambiguity inherent in monocular 3D pose estimation as multiple points can be projected onto the same point in an image. This ambiguity presents a significant challenge when attempting to reconstruct the 3D pose of a human using a single camera. As a consequence, angles of interest should be contained within the image plane as much as possible by orienting the camera accordingly or by performing the movements solely within the image plane. If this is not feasible due to setup constraints or the three-dimensionality of the movement of interest and high accuracy is essential, users are advised to use multi-view methods, which fuse the information of multiple cameras to achieve higher depth accuracy. If the use of monocular methods is unavoidable, users should consider using HPE methods with integrated kinematic body models, such as HSMR^75^, or methods, which consider time series information, such as MotionBERT, PoseFormerV2, and MotionAGFormer investigated in this study. It should be noted that these lifting methods are also capable of improving the accuracy of 2D poses, and therefore the use of such lifting methods may be beneficial even when a depth estimate is not required (Supplementary tables S3 and S4). Furthermore, if very high inference speeds are required, BlazePose is capable of providing accurate results in 2D when using the ’Local’ mode by discarding the depth estimates.

Although monocular HPE is not accurate enough to achieve clinically relevant measurements in its current state, it could be a viable alternative and improvement to visual assessments for applications where more accurate methods such as OMC are not feasible. When using HPE methods, the effect of inter-rater errors can be reduced, and bias when performing measurement post-intervention can be eliminated. The accuracy of monocular HPE could be further improved by avoiding measurements of partially occluded joint angles, specifying the posture and orientation of participants towards the camera to limit measurements in depth, and fine-tuning of models on application-specific datasets. Future studies might investigate the accuracy of HPE methods at measuring isolated joint flexion motions without self-occlusion. Future developments in HPE methods could aim to increase the accuracy of the joints and angles of the upper limb.

### Differences between HPE methods

The differences in accuracy between the investigated pose estimators could be explained by their different architectures and training datasets. Detectron2 is based on the Keypoint R-CNN architecture and the only investigated pose estimator, which was not purposefully designed for this task and therefore does not consider context and spatial relations when predicting joint locations, which resulted in its lower accuracy. The improved accuracy of HRNet over OpenPose and Alphapose could be explained by its parallel path architecture, whereby high-resolution features are retained throughout the network and are not reconstructed from low-resolution features. BlazePose shows high accuracy even though it does not employ such a parallel network and is the only pose estimator that performs direct joint regression. The accuracy could be explained by its training dataset, as it was trained on a dataset with a strong emphasis on yoga and fitness, with complex poses similar to our dataset. Furthermore, it uses an encoder that was trained on heatmap estimations. RTMPose and its variants RTMW and RTMO show the highest accuracy among the 2D pose estimators investigated. These are the only pose estimators that treat HPE as two independent classification tasks with subpixel precision and thereby reduce quantization errors. Furthermore, they retain spatial information and use transformers, which capture global and local spatial dependencies between joints. PoseFormerV2 shows slightly better accuracy than MotionBERT for most input sequences, possibly due to frequency filtering, which reduces noise and jitter. Note that MotionBERT shows lower errors within the image plane but higher errors in depth than PoseFormerV2. MotionAGFormer showed the highest accuracy among the investigated lifting methods, possibly due to its transformer and GCN layout. All transformers exhibited the effect of reducing in-plane errors for some input pose estimators, yet their depth estimation underperformed BlazePose, which is a direct pose estimator. MotionAGFormer and PoseFormerV2 furthermore displayed the ability to fill gaps within the input sequence, which is very useful for many applications. It should be noted that the training datasets varied between the investigated HPE methods. Some methods, like RTMPose, were trained on multiple datasets, whereas some were trained solely on a single dataset, and others were trained on nonpublic datasets. BlazePose was trained on a purpose-captured training dataset focusing on fitness and yoga. The poses in this dataset are highly similar to the ones encountered in our dataset, which could provide a further explanation for the good performance of BlazePose in our study. As HPE methods are most commonly used off-the-shelf, we determined their accuracy without further training or fine-tuning.

An effect of network architecture on the inference speed could also be noticed. Early two-stage methods like OpenPose, Detectron2, HRNet, and AlphaPose already achieved inference speeds over 15 FPS, which ensures viewer enjoyment and performance in real-time applications^76^. The speed of newer models, which avoid the computationally costly heatmap estimation, surpassed these. RTMPose, RTMW, and RTMO use the subpixel SimCC approach to HPE and do not require costly heatmaps, postprocessing or upsampling layers to achieve high accuracy. RTMO displayed especially high inference speeds as a result of being a one-stage method, which does not require a preprocessing step by a separate detector. BlazePose showed the highest inference speeds even though it is a two-stage method because it treats HPE as a regression task and its novel skip-frame face detector. This method uses the pose estimation results from a previous step to create the bounding box for the next frame. The detection step is only rerun when no human is visible, substantially increasing the inference speed. The investigated lifting methods generally show much higher inference speeds than the direct pose estimators, possibly due to the smaller input sizes. PoseFormerV2 displayed the lowest inference speed among the three lifting methods, possibly due to the discrete cosine transform of the input sequences. Even though MotionAGFormer lifts an entire input sequence in a single pass, it showed only about half the inference speed of MotionBERT. The lower speed of MotionAGFormer might be due to its dual stream architecture that includes a graph convolutional network.

Although lifting methods tend to be very fast, their real-time applications might be limited as they require a prior direct pose estimator to predict the input pose sequences. Larger receptive fields require longer input pose sequences, which will largely increase latency. This latency will increase drastically when choosing to use future frames to be included in the receptive field of the current frame. Although real-time applications might be limited, the very high speeds of lifting methods would still allow results to be available very shortly after capturing poses. Therefore, lifting methods might be more suitable for applications which do not require real-time results but might benefit from the increased accuracy of lifted pose sequences. For example, tracking rehabilitation progress or measuring range of motion requires high accuracy, and short delays for results to be available are often acceptable.

These findings emphasize the importance of network architecture and the capturing of spatial context in human pose estimation. Maintaining high-resolution features and leveraging spatial dependencies can improve accuracy. SimCC’s approach of treating HPE as two classification tasks reduces discretization errors from heatmap methods. Regression methods and training with heatmaps are another viable alternative for HPE. Transformers and attention mechanisms, which capture spatial relations, can further improve poses, but accuracy in depth shows potential for improvements. Training on application-specific datasets, as done with BlazePose, seems to be highly beneficial. Note that the accuracy of lifting methods is strongly dependent on the selected prior pose estimation step, with a range of $3D \ \overline{MPJPE}$ up to 40 mm being observable. These methods showed the ability to improve the in-plane accuracy of some 2D pose sequences, likely because they are able to capture temporal and spatial relations across frames. Their depth estimation underperformed BlazePose, possibly due to differences in their training datasets. The ’Local’ operating mode of BlazePose might be able to show even higher accuracy if a constant depth normalization factor is found.

### Limitations of study

One limitation of this study is the use of passive marker OMC as ground truth, as this measurement method displays errors compared to more accurate measurement methods such as fluoroscopy. The primary source of inaccuracy in OMC can be attributed to soft tissue artifacts, which are defined as the displacement of markers during movement^77^. As markers are assumed to be attached to a rigid body, deformations of the skin, tissue, and muscle may result in incorrect reconstruction of the joint centers and consequently of the joint angles^78^. The misplacement of markers is another source of errors^78^, which we tried to reduce by having two trained experts place the reflective markers.

The range of marker displacements due to soft tissue artifacts has been reported to be between 3 and 54 mm when using the Conventional Gait Model^79^, which we used in our study. These artifacts, in addition to the unconstrained segment lengths of the Conventional Gait Model, result in position errors of the joints. The precise magnitude of these position errors remains largely unknown for this model^80^. However, they have been estimated to be approximately 31 mm for the hip joint center on average^81^. These errors in joint center positions are then propagated towards the measured joint angles. For knee flexion, errors due to misplaced markers are estimated to be $0.9^{\circ }$, while the error due to soft tissue artifacts is estimated to be $3.3^{\circ }$ when using the Conventional Gait Model^78^. A further review revealed that the approximate error for knee flexion angles is less than $5^{\circ }$ in different 3D gait measurement methods^82^. Errors in the measurement of the angles in the upper body were found to be approximately $10^{\circ }$ when using the comparable Heidelberg Upper Extremity model^83^. It should be noted that there are other widely accepted marker sets in addition to the chosen one (Fig. 2), which could result in different positions for the ground truth joints. The limitations of the Conventional Gait Model have been extensively documented in the literature. The most relevant shortcomings of the model with respect to this study are the unconstrained segment lengths, the insufficient validation of the upper body model, and the lower accuracy in estimating the centers of the hip joints than alternative methods^80^. The Procrustes-aligned position errors $2D \ \overline{PAPE}$ of the hip joint ranged between 43-51 mm and are therefore up to twice as large as the hip joint position error for OMC. The $3D \ \overline{PAPE}$ of the hip joint ranged between 74-114 mm and is therefore three to four times larger than for OMC. We chose passive marker OMC and the Conventional Gait Model as the ground truth method due to their compatibility with the chosen exercises, their prevalence in clinical and research settings, and a lack of suitable non-intrusive alternatives. As the joint angle errors and hip joint errors of HPE methods are multiple times larger, passive marker OMC remains a valid ground truth reference when assessing the accuracy of HPE methods.

Another limitation is the method by which the accuracy metrics were determined in this study. The mean values were taken over all frames of the entire dataset, which therefore included all participants, exercises, and camera orientations. This is similar to how accuracy metrics are assessed on other datasets^54,55^, but it neglects certain details. The participants in this study consisted of young, healthy, and non-impaired individuals. For this reason, the insights of this study might not extend to other clinical use cases with impaired individuals, such as the analysis of pathological gait patterns. Furthermore, ethnicity was not considered in the recruitment of participants, which resulted in a homogeneous pool of participants. The appearance of participants may influence the accuracy of markerless pose estimators due to their training data. Ethnicity and appearance might impact the accuracy of markerless pose estimation methods, which was not investigated in this study.

Furthermore, the accuracy of all the pose estimators investigated is dependent on the orientation of the cameras and the type of exercise being performed. Due to self-occlusion and complex postures, the accuracy of monocular pose estimators can vary significantly between different camera orientations. Consequently, we determined the metrics by averaging over the entire dataset without considering self-occlusion and optimal viewing angles. This results in metrics that represent general accuracy and not the ideally achievable one. As the current study was captured in a laboratory setting with only one visible human, uniform lighting, minimal background interference, and because participants predominantly wore tight and short sports clothing, the results of this study might differ from the accuracy experienced in real-world applications. The use of HPE methods at home or in the wild might lead to lower accuracies due to the influence of lighting, occlusions, background, clothing, or multiple visible humans.

The largest limitation of existing monocular markerless pose estimators is their lack of accuracy in depth estimation. Although the overall 3D accuracy of these methods is currently inadequate for clinical interpretations, limiting their use to 2D applications with optimal camera orientations could potentially achieve the desired level of accuracy. These methods may be more advantageous in non-clinical settings such as rehabilitation, sports science, or home use, where error tolerance is greater.

Future developments may include improving depth estimation accuracy and creating new datasets to improve robustness to complex poses and self-occlusion. Using multiple cameras, with an emphasis on reducing their number to maintain the low-cost advantages of monocular techniques, is also possible. Collecting diverse datasets with 3D ground truth labels is crucial to develop more accurate and robust pose estimators with joint locations defined similarly to established methods.

## Conclusion

This study assessed the accuracy, precision, and inference speed of 11 different open source 2D and 3D monocular markerless pose estimators on a newly captured dataset, which focused on unimpaired individuals performing physical exercise. Joint center predictions and knee and elbow flexion angles were compared with passive marker optical motion capturing. Using this dataset, we evaluated and compared pose estimators to guide future users in their choice and to highlight the current limitations of such methods. The accuracy and precision varied greatly between the pose estimators and the image dimensions. Some 2D pose estimators were able to measure projected knee flexion angles with an accuracy comparable to or better than visual inspection. Currently, 3D pose estimators and transformer-based approaches do not deliver accurate depth estimations. However, markerless pose estimators offer advantages due to their affordability and minimal hardware needs. Furthermore, their accuracy is much greater when limiting use cases to 2D ones without depth estimations. These benefits may outweigh the drawbacks in many applications with high error tolerances. Human pose estimators can be a suitable alternative to visual inspections due to their comparable accuracy in certain tasks, reduced bias, and greater repeatability. They also offer benefits for applications that prioritize ease and cost, such as home therapy, remote assessments, and exercise supervision. Enhancing depth accuracy and capturing task-specific datasets as in this study could make human pose estimators a viable measurement method for more applications.

## Supplementary Information

Supplementary Information.

## Data Availability

All data generated during the current study are available from the corresponding author on reasonable request. The dataset analyzed during the current study is not publicly available due to privacy protection.
