# Supplementary material for: Assessment of monocular human pose estimation models for clinical movement analysis
Source: Sci Rep. 2025 Nov 5;15:38767. doi: 10.1038/s41598-025-22626-7 (PMC12589393; doi:10.1038/s41598-025-22626-7)
Supplement: Supplementary file 1 — Supplementary Information. [file 41598_2025_22626_MOESM1_ESM.pdf]

# Supplementary Information

**Supplementary table S1.** Accuracy metrics and inference speed of 2D pose estimators.

| Pose Estimator | $\overline{e_x}$ (mm) | $\overline{e_y}$ (mm) | 2D $\overline{MPJPE}$ (mm) | 2D $\overline{MAE}^{Knee}$ (degrees) | 2D $\overline{MAE}^{Elbow}$ (degrees) | Detected Poses (%) | Inference Speed (FPS) |
|---|---|---|---|---|---|---|---|
| OpenPose | 49 | 63 | 89 | 21.2 | 21.5 | 56.5 | 33 |
| AlphaPose | 46 | 62 | 86 | 17.7 | 25.9 | 99.6 | 37 |
| HRNet | 44 | 60 | 83 | 13.7 | 25.6 | 100.0 | 55 |
| Detectron2 | 51 | 65 | 93 | 18.0 | 27.8 | 99.8 | 25 |
| BlazePose World Lite | 55 | 66 | 97 | 21.9 | 26.1 | 98.7 | 134 |
| BlazePose World Full | 47 | 58 | 83 | 17.4 | 26.6 | 98.9 | 200 |
| BlazePose World Heavy | 46 | 58 | 83 | 15.5 | 25.5 | 98.7 | 147 |
| BlazePose Local Lite | 50 | 63 | 90 | 19.5 | 26.1 | 98.8 | 134 |
| BlazePose Local Full | 42 | 55 | 77 | 14.2 | 25.6 | 99.0 | 200 |
| BlazePose Local Heavy | 40 | 52 | 74 | 12.3 | 25.7 | 98.8 | 147 |
| RTMPose Lightweight | 73 | 79 | 122 | 16.2 | 28.9 | 100.0 | 131 |
| RTMPose Balanced | 45 | 55 | 80 | 11.2 | 27.6 | 100.0 | 62 |
| RTMPose Performance | 37 | 52 | 72 | 9.3 | 24.9 | 100.0 | 30 |
| RTMO Lightweight | 44 | 64 | 85 | 16.0 | 25.3 | 99.7 | 170 |
| RTMO Balanced | 42 | 56 | 78 | 13.4 | 23.4 | 99.7 | 111 |
| RTMO Performance | 38 | 51 | 72 | 10.6 | 24.4 | 99.9 | 74 |
| RTMW Lightweight | 47 | 67 | 92 | 14.8 | 27.2 | 100.0 | 88 |
| RTMW Balanced | 39 | 56 | 76 | 10.4 | 26.1 | 100.0 | 45 |
| RTMW Performance | 38 | 52 | 72 | 9.4 | 24.9 | 100.0 | 58 |

**Supplementary table S2.** Procrustes-aligned mean per joint and Procrustes-aligned joint-specific position errors of 2D pose estimators.

| Pose Estimator | 2D $\overline{PAMPJPE}$ (mm) | 2D $\overline{PAPE}^{Wrist}$ (mm) | 2D $\overline{PAPE}^{Elbow}$ (mm) | 2D $\overline{PAPE}^{Shoulder}$ (mm) | 2D $\overline{PAPE}^{Hip}$ (mm) | 2D $\overline{PAPE}^{Knee}$ (mm) | 2D $\overline{PAPE}^{Ankle}$ (mm) |
|---|---|---|---|---|---|---|---|
| OpenPose | 62 | 75 | 58 | 61 | 49 | 54 | 72 |
| AlphaPose | 62 | 68 | 62 | 62 | 46 | 57 | 76 |
| HRNet | 59 | 67 | 60 | 67 | 47 | 54 | 61 |
| Detectron2 | 67 | 75 | 68 | 69 | 50 | 61 | 79 |
| BlazePose World Lite | 68 | 69 | 64 | 61 | 51 | 74 | 88 |
| BlazePose World Full | 59 | 58 | 58 | 56 | 50 | 69 | 64 |
| BlazePose World Heavy | 60 | 61 | 61 | 66 | 43 | 67 | 62 |
| BlazePose Local Lite | 67 | 71 | 64 | 64 | 49 | 69 | 86 |
| BlazePose Local Full | 57 | 62 | 58 | 59 | 45 | 57 | 62 |
| BlazePose Local Heavy | 54 | 60 | 56 | 57 | 43 | 48 | 58 |
| RTMPose Lightweight | 74 | 93 | 81 | 77 | 50 | 62 | 81 |
| RTMPose Balanced | 59 | 74 | 64 | 60 | 45 | 45 | 65 |
| RTMPose Performance | 49 | 52 | 51 | 54 | 44 | 40 | 52 |
| RTMO Lightweight | 62 | 67 | 62 | 62 | 49 | 53 | 80 |
| RTMO Balanced | 55 | 62 | 57 | 55 | 46 | 46 | 66 |
| RTMO Performance | 51 | 54 | 53 | 53 | 44 | 41 | 59 |
| RTMW Lightweight | 61 | 67 | 64 | 63 | 47 | 55 | 67 |
| RTMW Balanced | 55 | 61 | 58 | 61 | 45 | 44 | 61 |
| RTMW Performance | 49 | 52 | 51 | 55 | 44 | 39 | 53 |

**Supplementary table S3.** Accuracy metrics and inference speed of 3D pose estimators. 2D values were computed by not considering the depth components.

| Pose Estimator | $\overline{e_x}$ (mm) | $\overline{e_y}$ (mm) | $\overline{e_z}$ (mm) | 2D $\overline{MPJPE}$ (mm) | 2D $\overline{MAE}^{Elbow}$ (degrees) | 2D $\overline{MAE}^{Knee}$ (degrees) | 3D $\overline{MPJPE}$ (mm) | 3D $\overline{MAE}^{Elbow}$ (degrees) | 3D $\overline{MAE}^{Knee}$ (degrees) | Detected Poses (%) | Inference Speed (FPS) |
|---|---|---|---|---|---|---|---|---|---|---|---|
| BlazePose World Lite | 55 | 66 | 111 | 97 | 26.1 | 21.9 | 162 | 17.8 | 22.2 | 98.7 | 134 |
| BlazePose World Full | 47 | 58 | 108 | 83 | 26.6 | 17.4 | 149 | 18.4 | 18.1 | 98.9 | 200 |
| BlazePose World Heavy | 46 | 58 | 105 | 83 | 25.5 | 15.5 | 146 | 18.0 | 17.2 | 98.7 | 147 |
| BlazePose Local Lite | 50 | 63 | 214 | 90 | 26.1 | 19.5 | 249 | 21.2 | 25.8 | 98.8 | 134 |
| BlazePose Local Full | 42 | 55 | 211 | 77 | 25.6 | 14.2 | 238 | 20.3 | 21.3 | 99.0 | 200 |
| BlazePose Local Heavy | 40 | 52 | 215 | 74 | 25.7 | 12.3 | 241 | 19.7 | 18.5 | 98.8 | 147 |
| MotionBERT on OpenPose | 38 | 65 | 128 | 83 | 27.6 | 18.6 | 167 | 25.0 | 14.1 | 14.4 | 1356 |
| MotionBERT on AlphaPose | 43 | 60 | 146 | 82 | 28.4 | 18.9 | 181 | 22.4 | 19.7 | 95.3 | 8877 |
| MotionBERT on HRNet | 42 | 61 | 146 | 83 | 31.2 | 15.4 | 182 | 22.5 | 19.1 | 100.0 | 9338 |
| MotionBERT on Detectron2 | 45 | 62 | 140 | 85 | 30.6 | 18.2 | 177 | 26.0 | 20.4 | 97.5 | 9095 |
| MotionBERT on BlazePose Local Lite | 44 | 60 | 121 | 83 | 28.3 | 16.0 | 161 | 23.3 | 17.8 | 85.3 | 7952 |
| MotionBERT on BlazePose Local Full | 39 | 55 | 123 | 74 | 27.9 | 12.1 | 156 | 21.4 | 15.5 | 89.6 | 8355 |
| MotionBERT on BlazePose Local Heavy | 37 | 52 | 119 | 72 | 27.9 | 10.3 | 151 | 21.5 | 14.3 | 88.4 | 8244 |
| MotionBERT on RTMPose Performance | 37 | 55 | 148 | 73 | 30.3 | 10.6 | 177 | 20.6 | 19.1 | 99.9 | 9341 |
| MotionBERT on RTMO Performance | 38 | 54 | 141 | 74 | 30.1 | 12.0 | 172 | 21.2 | 18.9 | 97.9 | 9160 |
| MotionBERT on RTMW Performance | 37 | 55 | 147 | 73 | 31.1 | 11.3 | 177 | 20.9 | 19.8 | 100.0 | 9027 |
| PoseFormerV2 on OpenPose | 47 | 69 | 118 | 94 | 21.2 | 19.3 | 167 | 17.2 | 22.0 | 100.0 | 120 |
| PoseFormerV2 on AlphaPose | 47 | 63 | 123 | 88 | 23.9 | 15.8 | 167 | 20.7 | 22.7 | 100.0 | 119 |
| PoseFormerV2 on HRNet | 48 | 65 | 127 | 90 | 26.8 | 14.5 | 172 | 21.3 | 19.3 | 100.0 | 121 |
| PoseFormerV2 on Detectron2 | 52 | 67 | 124 | 95 | 27.1 | 16.2 | 172 | 24.8 | 20.5 | 100.0 | 117 |
| PoseFormerV2 on BlazePose Local Lite | 48 | 60 | 119 | 87 | 23.7 | 15.0 | 162 | 18.9 | 22.2 | 100.0 | 117 |
| PoseFormerV2 on BlazePose Local Full | 43 | 57 | 122 | 79 | 24.1 | 11.0 | 160 | 17.7 | 20.3 | 100.0 | 117 |
| PoseFormerV2 on BlazePose Local Heavy | 41 | 55 | 115 | 76 | 23.7 | 9.7 | 152 | 17.5 | 18.3 | 100.0 | 121 |
| PoseFormerV2 on RTMPose Performance | 43 | 61 | 127 | 84 | 24.1 | 11.3 | 168 | 19.5 | 19.0 | 100.0 | 118 |
| PoseFormerV2 on RTMO Performance | 43 | 60 | 123 | 83 | 24.6 | 11.1 | 163 | 19.5 | 18.3 | 100.0 | 119 |
| PoseFormerV2 on RTMW Performance | 43 | 61 | 128 | 84 | 25.2 | 11.4 | 168 | 19.3 | 19.7 | 100.0 | 121 |
| MotionAGFormer on OpenPose | 52 | 59 | 151 | 89 | 18.8 | 15.9 | 193 | 20.3 | 20.5 | 100.0 | 4594 |
| MotionAGFormer on AlphaPose | 42 | 55 | 146 | 77 | 22.8 | 16.8 | 179 | 20.9 | 21.7 | 100.0 | 4602 |
| MotionAGFormer on HRNet | 42 | 57 | 146 | 79 | 24.7 | 14.1 | 180 | 20.1 | 19.3 | 100.0 | 4590 |
| MotionAGFormer on Detectron2 | 47 | 59 | 155 | 85 | 25.9 | 17.3 | 192 | 23.3 | 20.8 | 100.0 | 4573 |
| MotionAGFormer on BlazePose Local Lite | 41 | 50 | 123 | 73 | 22.3 | 14.7 | 157 | 17.3 | 20.3 | 100.0 | 4553 |
| MotionAGFormer on BlazePose Local Full | 36 | 46 | 121 | 66 | 22.7 | 11.2 | 151 | 16.8 | 19.0 | 100.0 | 4541 |
| MotionAGFormer on BlazePose Local Heavy | 34 | 43 | 119 | 61 | 22.1 | 8.5 | 146 | 16.3 | 16.7 | 100.0 | 4580 |
| MotionAGFormer on RTMPose Performance | 36 | 50 | 128 | 69 | 24.1 | 9.3 | 158 | 18.6 | 18.8 | 100.0 | 4559 |
| MotionAGFormer on RTMO Performance | 36 | 50 | 129 | 69 | 24.0 | 10.3 | 158 | 18.1 | 17.3 | 100.0 | 4573 |
| MotionAGFormer on RTMW Performance | 37 | 50 | 132 | 70 | 24.7 | 9.6 | 161 | 18.5 | 18.6 | 100.0 | 4575 |

**Supplementary table S4.** Procrustes-aligned mean per joint and Procrustes-aligned joint-specific position errors of 3D pose estimators. 2D values were computed by not considering the depth components.

| Pose Estimator | 2D $\overline{PAMPJPE}$ (mm) | 2D $\overline{PAPE}^{Wrist}$ (mm) | 2D $\overline{PAPE}^{Elbow}$ (mm) | 2D $\overline{PAPE}^{Shoulder}$ (mm) | 2D $\overline{PAPE}^{Hip}$ (mm) | 2D $\overline{PAPE}^{Knee}$ (mm) | 2D $\overline{PAPE}^{Ankle}$ (mm) | 3D $\overline{PAMPJPE}$ (mm) | 3D $\overline{PAPE}^{Wrist}$ (mm) | 3D $\overline{PAPE}^{Elbow}$ (mm) | 3D $\overline{PAPE}^{Shoulder}$ (mm) | 3D $\overline{PAPE}^{Hip}$ (mm) | 3D $\overline{PAPE}^{Knee}$ (mm) | 3D $\overline{PAPE}^{Ankle}$ (mm) |
|---|---|---|---|---|---|---|---|---|---|---|---|---|---|---|
| BlazePose World Lite | 68 | 69 | 64 | 61 | 51 | 74 | 88 | 114 | 150 | 101 | 92 | 82 | 115 | 141 |
| BlazePose World Full | 59 | 58 | 58 | 56 | 50 | 69 | 64 | 106 | 135 | 95 | 90 | 82 | 111 | 121 |
| BlazePose World Heavy | 60 | 61 | 61 | 66 | 43 | 67 | 62 | 107 | 129 | 102 | 99 | 75 | 110 | 125 |
| BlazePose Local Lite | 67 | 71 | 64 | 64 | 49 | 69 | 86 | 157 | 190 | 141 | 153 | 114 | 149 | 194 |
| BlazePose Local Full | 57 | 62 | 58 | 59 | 45 | 57 | 62 | 151 | 181 | 139 | 150 | 109 | 143 | 186 |
| BlazePose Local Heavy | 54 | 60 | 56 | 57 | 43 | 48 | 58 | 150 | 192 | 140 | 150 | 107 | 133 | 180 |
| MotionBERT on OpenPose | 56 | 67 | 54 | 64 | 44 | 42 | 68 | 95 | 130 | 88 | 92 | 74 | 76 | 112 |
| MotionBERT on AlphaPose | 66 | 76 | 65 | 66 | 51 | 59 | 79 | 137 | 184 | 124 | 112 | 95 | 126 | 180 |
| MotionBERT on HRNet | 64 | 76 | 66 | 73 | 50 | 56 | 61 | 136 | 186 | 127 | 117 | 91 | 124 | 171 |
| MotionBERT on Detectron2 | 66 | 78 | 68 | 70 | 50 | 57 | 75 | 135 | 185 | 128 | 115 | 87 | 119 | 175 |
| MotionBERT on BlazePose Local Lite | 67 | 84 | 68 | 63 | 52 | 60 | 77 | 116 | 165 | 105 | 99 | 84 | 105 | 138 |
| MotionBERT on BlazePose Local Full | 59 | 76 | 63 | 59 | 48 | 52 | 54 | 110 | 160 | 97 | 94 | 81 | 97 | 130 |
| MotionBERT on BlazePose Local Heavy | 57 | 76 | 64 | 58 | 47 | 45 | 51 | 107 | 155 | 96 | 93 | 82 | 91 | 122 |
| MotionBERT on RTMPose Performance | 55 | 64 | 57 | 62 | 48 | 45 | 54 | 135 | 185 | 122 | 117 | 88 | 116 | 182 |
| MotionBERT on RTMO Performance | 56 | 66 | 58 | 61 | 47 | 46 | 60 | 130 | 179 | 118 | 111 | 86 | 114 | 173 |
| MotionBERT on RTMW Performance | 55 | 64 | 57 | 63 | 47 | 45 | 55 | 135 | 184 | 122 | 118 | 88 | 119 | 179 |
| PoseFormerV2 on OpenPose | 61 | 64 | 42 | 78 | 61 | 44 | 75 | 100 | 120 | 67 | 100 | 88 | 95 | 129 |
| PoseFormerV2 on AlphaPose | 66 | 73 | 66 | 66 | 56 | 55 | 79 | 123 | 152 | 104 | 102 | 86 | 129 | 165 |
| PoseFormerV2 on HRNet | 67 | 75 | 68 | 77 | 56 | 57 | 70 | 124 | 160 | 111 | 111 | 88 | 122 | 153 |
| PoseFormerV2 on Detectron2 | 72 | 80 | 73 | 75 | 59 | 63 | 84 | 129 | 165 | 115 | 115 | 88 | 128 | 162 |
| PoseFormerV2 on BlazePose Local Lite | 66 | 72 | 62 | 67 | 53 | 57 | 83 | 120 | 147 | 102 | 107 | 77 | 120 | 163 |
| PoseFormerV2 on BlazePose Local Full | 59 | 68 | 59 | 64 | 52 | 48 | 62 | 117 | 145 | 100 | 106 | 77 | 118 | 158 |
| PoseFormerV2 on BlazePose Local Heavy | 56 | 65 | 57 | 62 | 52 | 43 | 58 | 110 | 139 | 92 | 100 | 77 | 110 | 143 |
| PoseFormerV2 on RTMPose Performance | 58 | 62 | 58 | 65 | 57 | 45 | 60 | 123 | 158 | 104 | 111 | 89 | 121 | 157 |
| PoseFormerV2 on RTMO Performance | 58 | 64 | 58 | 63 | 56 | 44 | 64 | 120 | 157 | 102 | 109 | 85 | 115 | 151 |
| PoseFormerV2 on RTMW Performance | 58 | 62 | 58 | 67 | 56 | 45 | 62 | 124 | 156 | 104 | 112 | 88 | 124 | 158 |
| MotionAGFormer on OpenPose | 49 | 50 | 37 | 54 | 47 | 39 | 66 | 100 | 135 | 76 | 89 | 90 | 97 | 112 |
| MotionAGFormer on AlphaPose | 58 | 63 | 56 | 56 | 45 | 53 | 73 | 126 | 170 | 121 | 107 | 88 | 125 | 147 |
| MotionAGFormer on HRNet | 57 | 63 | 57 | 64 | 48 | 51 | 59 | 129 | 178 | 126 | 116 | 91 | 127 | 137 |
| MotionAGFormer on Detectron2 | 62 | 68 | 62 | 63 | 49 | 55 | 76 | 138 | 185 | 136 | 122 | 92 | 130 | 161 |
| MotionAGFormer on BlazePose Local Lite | 57 | 60 | 53 | 53 | 45 | 55 | 75 | 112 | 148 | 103 | 96 | 82 | 112 | 133 |
| MotionAGFormer on BlazePose Local Full | 50 | 54 | 50 | 51 | 43 | 48 | 53 | 107 | 146 | 100 | 93 | 79 | 109 | 119 |
| MotionAGFormer on BlazePose Local Heavy | 46 | 52 | 48 | 48 | 41 | 39 | 49 | 105 | 145 | 99 | 94 | 78 | 102 | 111 |
| MotionAGFormer on RTMPose Performance | 48 | 51 | 49 | 53 | 45 | 40 | 51 | 114 | 154 | 101 | 107 | 86 | 112 | 123 |
| MotionAGFormer on RTMO Performance | 49 | 52 | 50 | 51 | 45 | 40 | 57 | 114 | 159 | 104 | 105 | 85 | 108 | 125 |
| MotionAGFormer on RTMW Performance | 49 | 50 | 50 | 54 | 45 | 40 | 53 | 117 | 160 | 106 | 111 | 87 | 114 | 125 |

**Supplementary table S5.** Overview of 3D $\overline{MPJPE}$ values from literature using Human3.6M and protocol 1 with subjects 9 and 11 for testing

| Year | Author | 3D $\overline{MPJPE}$ | Source |
|---|---|---|---|
| 2012 | Dai et al. | 136.9 | doi.org/10.1109/CVPR.2012.6247905, doi.org/10.48550/arXiv.1701.02354 |
| 2012 | Ramakrishna et al. | 157.3 | doi.org/10.48550/arXiv.1607.08128, doi.org/10.1007/978-3-642-33765-9_41, doi.org/10.48550/arXiv.1701.02354 |
| 2014 | Ionescu et al. | 127.9 | doi.org/10.1109/TPAMI.2013.248 |
| 2014 | Ionescu et al. | 150.7 | doi.org/10.1109/TPAMI.2013.248 |
| 2014 | Ionescu et al. | 172.1 | doi.org/10.1109/TPAMI.2013.248 |
| 2014 | Ionescu et al. | 138.8 | doi.org/10.1109/TPAMI.2013.248 |
| 2015 | Akhter and Black | 181.1 | doi.org/10.48550/arXiv.1607.08128, doi.org/10.48550/arXiv.1702.02258, doi.org/10.1109/CVPR.2015.7298751 |
| 2015 | Li et al. | 121.3 | doi.org/10.48550/arXiv.1508.06708 |
| 2015 | Li and Chan | 133.5 | doi.org/10.48550/arXiv.1508.06708 |
| 2016 | Zhou et al. | 113 | doi.org/10.48550/arXiv.1511.09439 |
| 2016 | Rogez et al. | 121.2 | doi.org/10.48550/arXiv.1607.02046 |
| 2016 | Sanzari et al. | 93.2 | doi.org/10.1007/978-3-319-46484-8_34 |
| 2016 | Tekin et al. | 147.7 | doi.org/10.48550/arXiv.1511.06692 |
| 2016 | Bogo et al. | 82.3 | doi.org/10.48550/arXiv.1607.08128 |
| 2016 | Du et al. | 126.5 | doi.org/10.1007/978-3-319-46493-0_2 |
| 2016 | Grinciunaite | 119 | doi.org/10.1007/978-3-319-49409-8_5 |
| 2016 | Park et al. | 117.3 | doi.org/10.48550/arXiv.1608.03075 |
| 2016 | Tekin et al. | 126 | doi.org/10.48550/arXiv.1511.06692 |
| 2016 | Zhou et al. | 107.3 | doi.org/10.48550/arXiv.1609.05317 |
| 2016 | Tekin et al. | 125 | doi.org/10.48550/arXiv.1511.06692 |
| 2016 | Yasin et al. | 108.3 | doi.org/10.48550/arXiv.1509.06720 |
| 2017 | Tung et al. | 79 | doi.org/10.48550/arXiv.1705.11166 |
| 2017 | Lassner et al. | 80.7 | doi.org/10.48550/arXiv.1701.02468 |
| 2017 | Chen and Ramanan | 114.2 | doi.org/10.48550/arXiv.1612.06524 |
| 2017 | Tung et al. | 97.2 | doi.org/10.48550/arXiv.1705.11166 |
| 2017 | Jahangiri et al. | 68 | doi.org/10.48550/arXiv.1702.02258 |
| 2017 | Coskun et al. | 71 | doi.org/10.48550/arXiv.1708.01885 |
| 2017 | Lin et al. | 73.1 | doi.org/10.48550/arXiv.1707.09695 |
| 2017 | Mehta et al. | 80.5 | doi.org/10.1145/3072959.3073596 |
| 2017 | Mehta et al. | 82.5 | doi.org/10.1145/3072959.3073596 |
| 2017 | Zhou et al. | 106.7 | doi.org/10.48550/arXiv.1607.08128, doi.org/10.48550/arXiv.1808.05942, doi.org/10.1109/TPAMI.2016.2605097 |
| 2017 | Lassner et al. | 82.3 | doi.org/10.48550/arXiv.1701.02468 |
| 2017 | Lassner et al. | 93.9 | doi.org/10.48550/arXiv.1701.02468 |
| 2017 | Nie et al. | 97.5 | doi.org/10.1109/ICCV.2017.373 |
| 2017 | Tekin et al. | 69.7 | doi.org/10.48550/arXiv.1611.05708 |
| 2017 | Rogez et al. | 87.7 | doi.org/10.1109/CVPR.2017.134 |
| 2017 | Sun et al. | 59.1 | doi.org/10.1109/ICCV.2017.284 |
| 2017 | Martinez et al. | 62.9 | doi.org/10.48550/arXiv.1705.03098 |
| 2017 | Tome et al. | 88.4 | doi.org/10.1109/CVPR.2017.603 |
| 2017 | Sun et al. | 92.4 | doi.org/10.1109/ICCV.2017.284 |
| 2017 | Martinez et al. | 45.5 | doi.org/10.48550/arXiv.1705.03098 |
| 2017 | Mehta et al. | 72.9 | doi.org/10.48550/arXiv.1611.09813 |
| 2017 | Moreno-Noguer | 87.3 | doi.org/10.1109/CVPR.2017.170 |
| 2017 | Zhou et al. | 64.9 | doi.org/10.48550/arXiv.1704.02447 |
| 2017 | Pavlakos et al. | 71.9 | doi.org/10.1109/CVPR.2017.139 |
| 2018 | Varol et al. | 49 | doi.org/10.48550/arXiv.1804.04875 |
| 2018 | Omran | 59.9 | doi.org/10.48550/arXiv.1808.05942 |
| 2018 | ZhaoXiaowei et al. | 77.8 | doi.org/10.48550/arXiv.1701.02354 |
| 2018 | Pavlakos et al. | 75.9 | doi.org/10.48550/arXiv.1805.04092 |
| 2018 | ZhaoXiaowei et al. | 79.6 | doi.org/10.48550/arXiv.1701.02354 |
| 2018 | Yang et al. | 58.6 | doi.org/10.48550/arXiv.1803.09722 |
| 2018 | Rogez et al. | 110.6 | doi.org/10.1007/s11263-018-1071-9 |
| 2018 | ZhouXiaowei et al. | 77.3 | doi.org/10.48550/arXiv.1701.02354 |
| 2018 | Katircioglu et al. | 127.1 | doi.org/10.1007/s11263-018-1066-6 |
| 2018 | Katircioglu et al. | 67.3 | doi.org/10.1007/s11263-018-1066-6 |
| 2018 | Katircioglu et al. | 66.2 | doi.org/10.1007/s11263-018-1066-6 |
| 2018 | Katircioglu et al. | 91.6 | doi.org/10.1007/s11263-018-1066-6 |
| 2018 | Katircioglu et al. | 66 | doi.org/10.1007/s11263-018-1066-6 |
| 2018 | Katircioglu et al. | 65.4 | doi.org/10.1007/s11263-018-1066-6 |
| 2018 | Sarandi et al. | 56.1 | doi.org/10.48550/arXiv.1808.09316 |
| 2018 | Luo et al. | 63.7 | doi.org/10.48550/arXiv.1811.04989 |
| 2018 | ZhouXiaowei et al. | 90.2 | doi.org/10.1109/ICRA.2018.8462830, doi.org/10.48550/arXiv.1701.02354 |

Continued on next page

**Continued from previous page**

| Year | Author | 3D $\overline{MPJPE}$ | Source |
|---|---|---|---|
| 2018 | Mehta et al. | 69.9 | doi.org/10.48550/arXiv.1712.03453 |
| 2018 | Luvizon et al. | 53.2 | doi.org/10.48550/arXiv.1802.09232 |
| 2018 | Fang et al. | 60.4 | doi.org/10.48550/arXiv.1710.06513 |
| 2018 | Sun et al. | 56.2 | doi.org/10.48550/arXiv.1711.08229 |
| 2018 | Sun et al. | 59.3 | doi.org/10.48550/arXiv.1711.08229 |
| 2018 | Sun et al. | 49.6 | doi.org/10.48550/arXiv.1711.08229 |
| 2018 | Sun et al. | 68 | doi.org/10.48550/arXiv.1711.08229 |
| 2018 | Sun et al. | 64.1 | doi.org/10.48550/arXiv.1711.08229 |
| 2018 | Kanazawa et al. | 88 | doi.org/10.48550/arXiv.1712.06584 |
| 2018 | Dabral et al. | 55.5 | doi.org/10.48550/arXiv.1711.09250 |
| 2018 | Dabral et al. | 52.1 | doi.org/10.48550/arXiv.1711.09250 |
| 2018 | Hossain et al. | 51.9 | doi.org/10.1007/978-3-030-01249-6_5 |
| 2018 | Mehta et al. | 84.3 | doi.org/10.48550/arXiv.1712.03453 |
| 2018 | Hossain et al. | 39.2 | doi.org/10.1007/978-3-030-01249-6_5 |
| 2018 | Luvizon et al. | 55.1 | doi.org/10.48550/arXiv.1802.09232 |
| 2018 | Rhodin et al. | 66.8 | doi.org/10.48550/arXiv.1803.04775 |
| 2018 | Lee et al. | 55.8 | doi.org/10.1007/978-3-030-01234-2_8 |
| 2018 | Lee et al. | 40.9 | doi.org/10.1007/978-3-030-01234-2_8 |
| 2018 | Lee et al. | 38.4 | doi.org/10.1007/978-3-030-01234-2_8 |
| 2018 | Pavlakos et al. | 56.2 | doi.org/10.1109/CVPR.2018.00763 |
| 2019 | Cheng et al. | 42.9 | doi.org/10.1109/ICCV.2019.00081 |
| 2019 | Pavllo et al. | 51.6 | doi.org/10.48550/arXiv.1811.11742 |
| 2019 | Liu et al. | 61.1 | doi.org/10.48550/arXiv.1901.04877 |
| 2019 | Cai et al. | 48.8 | doi.org/10.1109/ICCV.2019.00236 |
| 2019 | Arnab et al. | 77.8 | doi.org/10.48550/arXiv.1905.04266 |
| 2019 | Cai et al. | 50.6 | doi.org/10.1109/ICCV.2019.00236 |
| 2019 | Wandt and Rosenhahn | 89.9 | doi.org/10.1109/CVPR.2019.00797 |
| 2019 | Wandt and Rosenhahn | 50.9 | doi.org/10.1109/CVPR.2019.00797 |
| 2019 | Xu et al. | 76.8 | doi.org/10.48550/arXiv.1910.00116 |
| 2019 | Cai et al. | 49.1 | doi.org/10.1109/ICCV.2019.00236 |
| 2019 | Pavllo et al. | 51.8 | doi.org/10.48550/arXiv.1811.11742 |
| 2019 | Pavllo et al. | 37.2 | doi.org/10.48550/arXiv.1811.11742 |
| 2019 | Pavllo et al. | 53.4 | doi.org/10.48550/arXiv.1811.11742 |
| 2019 | Li et al. | 52.7 | doi.org/10.48550/arXiv.1904.05547 |
| 2019 | Pavllo et al. | 46.8 | doi.org/10.48550/arXiv.1811.11742 |
| 2019 | Zhao et al. | 60.8 | doi.org/10.1109/CVPR.2019.00354 |
| 2019 | Zhao et al. | 57.6 | doi.org/10.1109/CVPR.2019.00354 |
| 2019 | Zhao et al. | 43.8 | doi.org/10.1109/CVPR.2019.00354 |
| 2019 | Sharma et al. | 58 | doi.org/10.48550/arXiv.1904.01324 |
| 2019 | Sharma et al. | 52.1 | doi.org/10.48550/arXiv.1904.01324 |
| 2019 | Wang et al. | 52.6 | doi.org/10.48550/arXiv.1905.07862 |
| 2019 | Habibie et al. | 65.7 | doi.org/10.1109/CVPR.2019.01116 |
| 2019 | Zhou et al. | 39.9 | doi.org/10.48550/arXiv.1910.12032 |
| 2019 | Rogez et al. | 61.2 | doi.org/10.1109/TPAMI.2019.2892985 |
| 2019 | Kolotouros et al. | 41.1 | doi.org/10.48550/arXiv.1909.12828 |
| 2019 | Moon et al. | 53.3 | doi.org/10.48550/arXiv.1907.11346 |
| 2019 | Moon et al. | 54.4 | doi.org/10.48550/arXiv.1907.11346 |
| 2019 | Rogez et al. | 63.5 | doi.org/10.1109/TPAMI.2019.2892985 |
| 2020 | Mehta et al. | 63.6 | doi.org/10.1145/3386569.3392410 |
| 2020 | Mitra et al. | 94.2 | doi.org/10.48550/arXiv.1908.05293 |
| 2020 | Fabbri et al. | 61 | doi.org/10.1109/CVPR42600.2020.00723 |
| 2020 | Chen et al. | 47.3 | doi.org/10.1007/978-3-030-58580-8_42 |
| 2020 | Liu et al. | 34.7 | doi.org/10.1109/CVPR42600.2020.00511 |
| 2020 | Liu et al. | 46.7 | doi.org/10.1109/CVPR42600.2020.00511 |
| 2020 | Xu et al. | 45.6 | doi.org/10.1109/CVPR42600.2020.00098 |
| 2020 | Xu et al. | 46.3 | doi.org/10.1109/CVPR42600.2020.00098 |
| 2020 | Xu et al. | 49.2 | doi.org/10.1109/CVPR42600.2020.00098 |
| 2020 | Liu et al. | 45.1 | doi.org/10.1109/CVPR42600.2020.00511 |
| 2021 | Zeng et al. | 47.9 | doi.org/10.1007/s10462-024-11019-3 |
| 2ís021 | Liu et al. | 44.8 | doi.org/10.1007/s10462-024-11019-3 |
| 2021 | Zou and Tang | 49.4 | doi.org/10.1007/s10462-024-11019-3 |
| 2021 | Xu et al. | 85.3 | doi.org/10.1007/s10462-024-11019-3 |
| 2021 | Wandt et al. | 81.9 | doi.org/10.1007/s10462-024-11019-3 |
| 2022 | Kundu et al. | 103.2 | doi.org/10.1007/s10462-024-11019-3 |
| 2022 | Xu et al. | 58.1 | doi.org/10.1007/s10462-024-11019-3 |
| 2022 | Kundu et al. | 59.4 | doi.org/10.1109/CVPR52688.2022.01980 |

Continued on next page

**Continued from previous page**

| Year | Author | 3D $\overline{MPJPE}$ | Source |
|---|---|---|---|
| 2022 | Kundu et al. | 103.2 | doi.org/10.1109/CVPR52688.2022.01980 |
| 2022 | Xu et al. | 58.1 | doi.org/10.1109/TPAMI.2021.3087695 |
| 2023 | Chai et al. | 49.9 | doi.org/10.1007/s10462-024-11019-3 |
| 2023 | Nie et al. | 50.3 | doi.org/10.1007/s10462-024-11019-3 |
| 2023 | Zhao et al. | 45.2 | doi.org/10.1007/s10462-024-11019-3 |
| 2023 | Tang et al. | 40.5 | doi.org/10.1007/s10462-024-11019-3 |
| 2023 | Honari et al. | 100.3 | doi.org/10.1007/s10462-024-11019-3 |
| 2023 | Yu et al. | 44.4 | doi.org/10.1007/s10462-024-11019-3 |
| 2023 | Li and Pun | 47.3 | doi.org/10.1007/s10462-024-11019-3 |
| 2023 | Lee et al. | 40.5 | doi.org/10.1007/s10462-024-11019-3 |
| 2023 | Luvizon et al. | 51.6 | doi.org/10.1016/j.patcog.2023.109714 |
| 2023 | Zhai et al. | 41.1 | doi.org/10.1007/s10462-024-11019-3 |
| 2023 | Chen et al. | 40.3 | doi.org/10.1007/s10462-024-11019-3 |
| 2023 | Gong et al. | 36.9 | doi.org/10.1007/s10462-024-11019-3 |
| 2023 | Li et al. | 42.5 | doi.org/10.1007/s10462-024-11019-3 |
| 2023 | Zhang et al. | 49.1 | doi.org/10.1007/s10462-024-11019-3 |
| 2023 | Luvizon et al. | 55.5 | doi.org/10.1007/s10462-024-11019-3 |
| 2023 | Kang et al. | 46.3 | doi.org/10.1007/s10462-024-11019-3 |
| 2024 | Zhou et al. | 42.6 | doi.org/10.1007/s10462-024-11019-3 |
| 2024 | Kim et al. | 69 | doi.org/10.1007/s10462-024-11019-3 |
| 2024 | Cai et al. | 39.7 | doi.org/10.1007/s10462-024-11019-3 |
| 2024 | Chen et al. | 49.8 | doi.org/10.1007/s10462-024-11019-3 |
